# The Disordered Region of ASXL1 Acts as an Auto‐Regulator Through Condensation

**DOI:** 10.1002/advs.202510999

**Published:** 2026-01-20

**Authors:** Xiao Fang, Qiwei Li, Wenqing Zhang

**Affiliations:** ^1^ Department of Hematology Guangzhou First People's Hospital School of Medicine South China University of Technology Guangzhou China; ^2^ Department of Hematology the Sixth Affiliated Hospital of South China University of Technology (Nanhai District People's Hospital of Foshan) Foshan China; ^3^ School of Biology and Biological Engineering South China University of Technology Guangzhou China; ^4^ Division of Cell Developmental and Integrative Biology School of Medicine South China University of Technology Guangzhou China

**Keywords:** ASXL1, auto‐regulation, BRD2, chromatin accessibility, liquid‐liquid phase separation

## Abstract

Intrinsically disordered regions (IDRs) are common in chromatin regulators, yet how their sequence encodes regulatory logic remains unclear. Here, we show that the long linker IDR of ASXL1 (Additional Sex Combs Like 1) functions as an embedded autoregulatory module. A basic condensation‐prone segment is suppressed by a downstream acidic “charge block,” forming an electrostatic switch that gates condensation. Disease‐associated truncations remove this inhibition, unleashing phase separation and recruiting BRD2 (Bromodomain‐containing protein 2) to ectopic chromatin loci. Distinct truncation sites yield graded effects on condensate formation, chromatin accessibility, and neutrophil differentiation. Charge‐reversing mutations restore liquid‐liquid phase separation (LLPS) in a sequence‐dependent manner. Proteomic and imaging analyses identify BRD2 as a key condensate‐integrated factor whose mislocalization alters chromatin state. A compound screen reveals that Tosedostat reduces C‐terminally truncated ASXL1 (ASXL1‐TR) condensation and partially restores nuclear segmentation. Together, these findings define a tunable electrostatic switch within a long IDR and establish a broader model in which autoregulatory IDRs orchestrate condensation, chromatin engagement, and lineage fidelity.

## Graphical abstract

ASXL1's long IDR encodes an electrostatic “basic platform + acidic brake” that autoregulates condensation. Truncation at a clinical hotspot lifts this brake, forming condensates that retarget BRD2, remodel local chromatin accessibility, and impair neutrophil maturation. Conversely, a long truncation containing AA718–918 retains the inhibitory segment and suppresses condensation. These results illustrate how charge‐patterned IDRs govern nuclear organization and lineage fidelity.

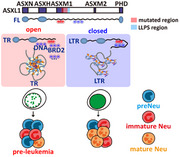

## 1 Introduction

Intrinsically disordered regions (IDRs) comprise ∼37%—50% of the human proteome [1] and play essential roles as flexible linkers, multivalent interaction hubs, sensors, and drivers of subcellular organization [2]. While most IDRs span 30—200 residues, a distinct subset exceeds 1000 amino acids [3]. When such extended regions lie between structured domains, we refer to them as long linker IDRs (llIDRs). Linker IDRs are often found in allosteric proteins, where they help coordinate interdomain communication [4, 5, 6]. The evolutionary rationale for preserving such long disordered segments—whether to increase mechanical reach, encode regulatory motifs, or facilitate phase behavior—remains poorly understood.

One prominent example is ASXL1, a chromatin scaffold frequently mutated in hematologic malignancies [7]. Pathogenic ASXL1 variants cluster within its llIDR and typically generate C‐terminal truncations [7]. Truncated ASXL1 proteins are detectable in myelodysplastic syndrome (MDS) cell lines [8] and often accumulate to higher levels than full‐length ASXL1 [9]. Recurrent truncating mutations cluster within a hotspot spanning ∼amino acids 591—693 [10], with additional sites elsewhere. Intriguingly, truncations at more distal sites (e.g., residue 957) yield gene expression profiles that are intermediate between wild‐type and more proximal truncations [11], suggesting region‐specific regulatory embedded within the llIDR.

ASXL1 is a multi‐domain chromatin regulator with an N‐terminal ASXN DNA‐binding domain [12] and a C‐terminal PHD (plant homeodomain) finger predicted to recognize methylated histone tails [13]. It engages multiple chromatin‐modifying complexes and transcriptional regulators, including PR‐DUB (Polycomb repressive deubiquitinase)/BAP1 (BRCA1 associated protein‐1) and others [7, 14, 15, 16, 17, 18]. Loss of its C‐terminal IDR has been shown to differentially affect cofactor binding, enhancing some interactions while disrupting others [9, 18, 19]. Recent work has implicated ASXL1 in phase separation [20], but the underlying regulatory logic—and how different truncations affect its biochemical and functional states—remains unresolved.

Here, we use ASXL1 as a model to dissect how long IDRs encode regulatory logic for nuclear condensation and chromatin control. We show that its llIDR contains discrete subregions that either promote or inhibit condensation via opposing electrostatic charges. These elements function as an autoregulatory switch: an inhibitory acidic segment suppresses a basic condensation‐prone domain through intramolecular antagonism. Patient‐derived truncations remove this inhibition, unleashing condensate formation, redirecting BRD2 chromatin occupancy, and impairing neutrophil differentiation. Together, our findings reveal a tunable electrostatic switch within a long IDR and propose a generalizable mechanism by which IDRs encode autoregulatory control over nuclear organization and cell fate.

## 2 Results

### 2.1 C‐Terminal Truncation of ASXL1 Promotes Nuclear Localization and Condensate Formation

**FIGURE 1:**
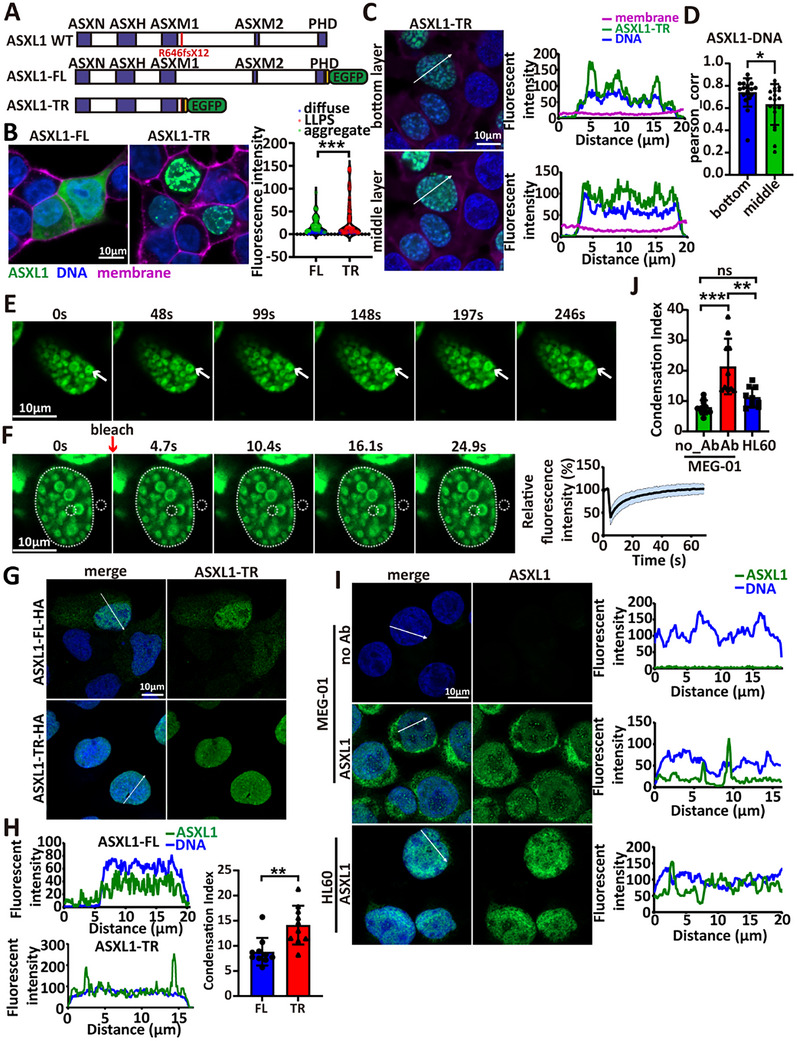
ASXL1‐TR exhibits enhanced nuclear localization and condensate formation. (A) Schematic of mEGFP‐tagged ASXL1 constructs linked via a cleavage‐deficient HA‐TEV‐mutated P2A sequence. (B) Representative confocal images of mEGFP‐tagged ASXL1‐FL and ASXL1‐TR in live 293T cells (field shown reflects moderate ASXL1‐FL expression, where FL is largely diffuse). Right: Violin plot quantifying condensate formation per cell; points are single cells, colored by classification (LLPS = red; aggregate = green; diffuse = blue); points are single cells (FL, *n* = 87; TR, *n* = 69). Aggregate‐class datapoints for FL correspond to cells at higher expression that form irregular, non‐recovering puncta (see Figure S2A,B); LLPS vs aggregate assignment is based on morphology and FRAP behavior (see Experimental Section). Statistics: *p*‐value < 2.2 × 10^−16^ and *p*‐value < 0.01 (ASXL1‐FL aggregates classified as condensates), ^*^ *p* < 0.05, ^**^ *p* < 0.01, ^***^ *p* < 0.001; mean ± SD. (C) Representative images and line‐scan quantifications of ASXL1‐TR‐mEGFP at the bottom and middle nuclear planes. (D) Pearson correlation between ASXL1‐TR and Hoechst; colocalization by FIJI/Coloc2 (FL, *n* = 19; TR, *n* = 19). Statistics: two‐tailed Student's *t*‐test (^*^ *p* < 0.05, ^**^ *p* < 0.01, ^***^ *p* < 0.001; mean ± SD). (E) Time‐lapse images showing fusion of ASXL1‐TR‐mEGFP condensates (white arrows mark droplets before and after fusion). (F) FRAP of ASXL1‐TR condensates. Left, representative images; right, fluorescence‐recovery curves. White dotted circles denote the bleached and background region; dashed outlines the total fluorescence area. *n* = 5 biological replicates. (G) Representative immunofluorescence of HA‐tagged ASXL1 in U2OS cells. (H) Line‐scan traces (left) and condensation Index (CI; right) from nuclear line‐scans (see Experimental Section) quantify puncta prominence in HA‐tagged U2OS cells (FL, *n* = 10; TR, *n* = 10). Statistics: two‐tailed Student's *t*‐test (^*^ *p* < 0.05, ^**^ *p* < 0.01, ^***^ *p* < 0.001; mean ± SD). (I) Immunofluorescence for endogenous ASXL1 in MEG‐01 (heterozygous truncation); HL‐60 (ASXL1‐FL) and no‐primary antibody (no Ab) are negative controls. (J) CI from nuclear line‐scans quantifies puncta prominence (no_Ab, *n* = 10; Ab, *n* = 10; HL‐60, *n* = 10). One‐way ANOVA with post‐hoc testing (^*^ *p* < 0.05, ^**^ *p* < 0.01, ^***^ *p* < 0.001; error bars: mean ± SD).

To investigate how the C‐terminal long linker IDR (llIDR) regulates ASXL1 behavior, we compared full‐length ASXL1 (ASXL1‐FL) with a common truncated mutant (ASXL1‐TR, R646fsX12) [10]. When tagged at residue 646 with mEGFP, ASXL1‐TR formed discrete nuclear puncta, whereas ASXL1‐FL remained diffusely distributed in 293T cells (Figure 1A,B). ASXL1‐TR puncta localized to the nuclear and nucleolar periphery and colocalized with Hoechst‐stained DNA (Figure 1C–D). Live‐cell imaging revealed dynamic droplet fusion and fission (Figure 1E and Movie S1), and Fluorescence recovery after photobleaching (FRAP) demonstrated rapid signal recovery, consistent with liquid‐like properties (Figure 1F and Movie S2).

To assess robustness, we expressed ASXL1‐FL and ASXL1‐TR in U2OS, HeLa and K562 cells. ASXL1‐TR consistently formed nuclear condensates across all tested cell types, which all had a higher condensation index (CI, see Experimental Section) than ASXL1‐FL (Figure S1A). To control for tag artifacts, we tested several C‐terminal tags (Figure S1B). ASXL1‐TR‐TagBFP and ASXL1‐TR‐mCherry formed condensates, although mCherry showed less defined boundaries and yielded the lowest CI and peak‐to‐valley contrast (PVR; see Experimental Section) (Figure S1B), consistent with its known solubilizing effect [21]. ASXL1‐TR‐mRuby did not form round puncta (Figure S1B, red arrow), possibly owing to its delayed maturation [22]. Among fluorescent proteins, TagBFP most closely recapitulated mEGFP, although it exhibited higher PVR. These data indicate that fluorescent protein fusions can influence condensate formation. Therefore, we further validated with an HA tag: ASXL1‐TR‐HA still formed nuclear condensates detectable by immunofluorescence (Figure 1G,H). We further assessed endogenous ASXL1 localization in MEG‐01, a hematopoietic (myeloid‐lineage) cell line harboring a heterozygous ASXL1 truncation. Using “no‐primary” and HL‐60 (ASXL1‐FL) as negative controls, we observed discrete nuclear puncta in MEG‐01 (Figure 1I,J).

A prior study reported that ASXL1‐WT forms condensates, whereas a truncation mutant at residue 635 does not, suggesting the C‐terminal IDR promotes liquid‐liquid phase separation (LLPS) [20]. Since that study used N‐terminally tagged constructs in HeLa cells, we asked whether tag orientation affects behavior. N‐terminally tagged ASXL1‐TR formed fewer condensates (Figure S1C) and showed reduced DNA association (Figure S1D), suggesting that N‐terminal tags interfere with DNA‐binding by the ASXN domain [12].

Interestingly, at high expression levels, ASXL1‐FL formed irregular cytoplasmic aggregates (non‐spherical puncta; Figure 1B and Figure S2A) that failed to recover after photobleaching (Figure S2B), consistent with an aggregation‐like, non‐liquid state, yet indicating an intrinsic self‐association propensity that is normally restrained. Most ASXL1‐FL remained cytoplasmic in our system, likely due to insufficient nuclear import machinery [9], leading to elevated cytoplasmic concentration and aggregation. When co‐expressed, ASXL1‐FL was incorporated into ASXL1‐TR condensates (Figure S2C), suggesting that, under permissive conditions, ASXL1‐FL can form condensates similar to those of ASXL1‐TR.

Together, these findings show that C‐terminal truncation promotes ASXL1 nuclear condensation by releasing autoinhibitory constraints.

### 2.2 C‐Terminal Truncations Within AA619–AA718 Facilitate ASXL1 Condensate Formation

**FIGURE 2:**
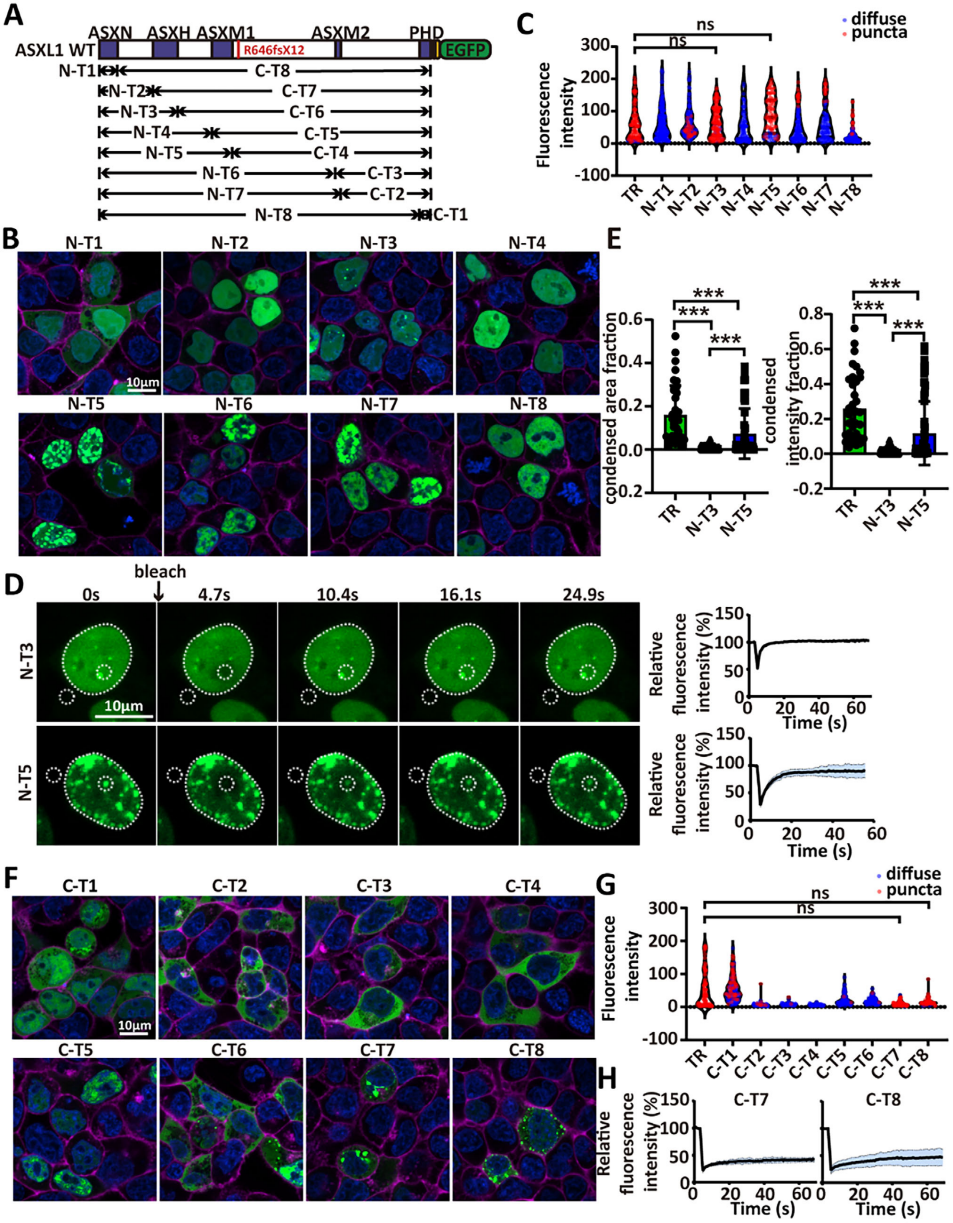
ASXH and ASXM1 domains are crucial for condensate formation. (A) Schematic of mEGFP‐tagged ASXL1 truncation constructs: C‐terminal (N‐T) and N‐terminal (C‐T) variants. (B) Confocal images of mEGFP‐tagged ASXL1 N‐T mutants (N‐Ts) in live 293T cells. (scale bar: 10 µm) (C) Violin plot quantifying condensate formation for each N‐T construct. Each point represents a single cell, with fluorescence intensity on the *y*‐axis and puncta presence (red) or absence (blue) indicated by color (TR, *n* = 104; N‐T1, *n* = 190; N‐T2, *n* = 124; N‐T3, *n* = 139; N‐T4, *n* = 130; N‐T5, *n* = 93; N‐T6, *n* = 110; N‐T7, *n* = 96; N‐T8, *n* = 104). For statistical analysis, we used the ASXL1‐TR as positive control. The resulting *p*‐value when each truncation was compared with ASXL1‐TR were: N‐T1 < 2.2 × 10^−16^; N‐T2 < 2.2 × 10^−16^; N‐T3 = 1; N‐T4 < 2.2 × 10^−16^; N‐T5 = 0.08917; N‐T6 < 2.2 × 10^−16^; N‐T7 < 2.2 × 10^−16^; N‐T8 = 3.08 × 10^−13^. (D) Representative FRAP images (left) and corresponding fluorescence recovery curves (right) for N‐T3 and N‐T5. White dotted circles denote bleached and background ROIs; the dashed line outlines the total fluorescence area. n = 5 biological replicates. (E) Condensed burden quantified as condensed area fraction (left) and condensed intensity fraction (right) per nucleus from confocal images (expression‐matched; see Experimental Section) (TR, *n* = 37; N‐T3, *n* = 81; N‐T5, *n* = 62). Statistics: one‐way ANOVA with post‐hoc testing (^*^ *p* < 0.05, ^**^ *p* < 0.01, ^***^ *p* < 0.001; error bars: mean ± SD.) (F) Confocal images of mEGFP‐tagged ASXL1 C‐T in live 293T cells. (G) Violin plot quantifying condensate formation for each C‐T construct, analyzed in the same manner as the N‐T constructs described above (TR, *n* = 90; C‐T1, *n* = 121; C‐T2, *n* = 66; C‐T3, *n* = 40; C‐T4, *n* = 35; C‐T5, *n* = 64; C‐T6, *n* = 68; C‐T7, *n* = 66; C‐T8, *n* = 47). When each truncation was compared with ASXL1‐TR, the resulting *p*‐values were: C‐T1 = 3.765 × 10^−14^; C‐T2 = 1.231 × 10^−14^; C‐T3 = 7.499 × 10^−13^; C‐T4 = 7.583 × 10^−15^; C‐T5 < 2.2 × 10^−16^; C‐T6 < 2.2 × 10^−16^; C‐T7 = 0.05798; C‐T8 = 0.1124. (H) Representative FRAP recovery curves for C‐T7 and C‐T8. *n* = 5 biological replicates.

To pinpoint ASXL1 regions responsible for condensation, we generated a series of mEGFP‐tagged truncation mutants and evaluated their subcellular localization and condensate‐forming capacity in 293T cells (Figure  2A). ASXL1‐TR served as a positive control, and expression levels were matched for comparison.

Among C‐terminal truncations (N‐Ts), only N‐T3 (retaining ASXH) and N‐T5 (extending through ASXM1) formed condensates (Figure  2B–C), which exhibited liquid‐like recovery by FRAP (Figure  2D). We quantified per‐cell condensed burden from confocal images, within expression‐matched bins, as condensed area fraction (Σ segmented foci area / nuclear area) and condensed intensity fraction (Σ segmented foci integrated fluorescence / total nuclear fluorescence) (see Experimental Section). Both metrics yielded the same ranking, TR > N‐T5 > N‐T3, with significant pairwise differences (Figure 2D). Condensate counts are provided for completeness and follow the same trend (Figure S3A). Together, these results indicate that ASXH and ASXM1 are necessary but not sufficient to achieve TR‐level condensation. A short segment immediately downstream of ASXM1 (aa 619–646) further enhances ASXL1's condensation capacity.

We next examined N‐terminal truncations (C‐Ts) retaining different C‐terminal regions (Figure  2A). Most constructs localized to the cytoplasm, except C‐T5 (which includes ASXM1) and C‐T1 (small enough to enter nuclei passively [23]), reinforcing a role for ASXM1 in nuclear import. C‐T7 and C‐T8, which contain ASXH and the upstream linker L1, formed cytoplasmic puncta (Figure  2F,G) that failed to recover in FRAP (Figure  2H and Figure S3B), consistent with aggregation rather than LLPS. Together, these results suggest that while the N‐terminal ASXH and L1 modules are required for self‐association, the C‐terminal region of ASXL1 can limit nuclear entry and suppress LLPS‐like dynamics.

**FIGURE 3:**
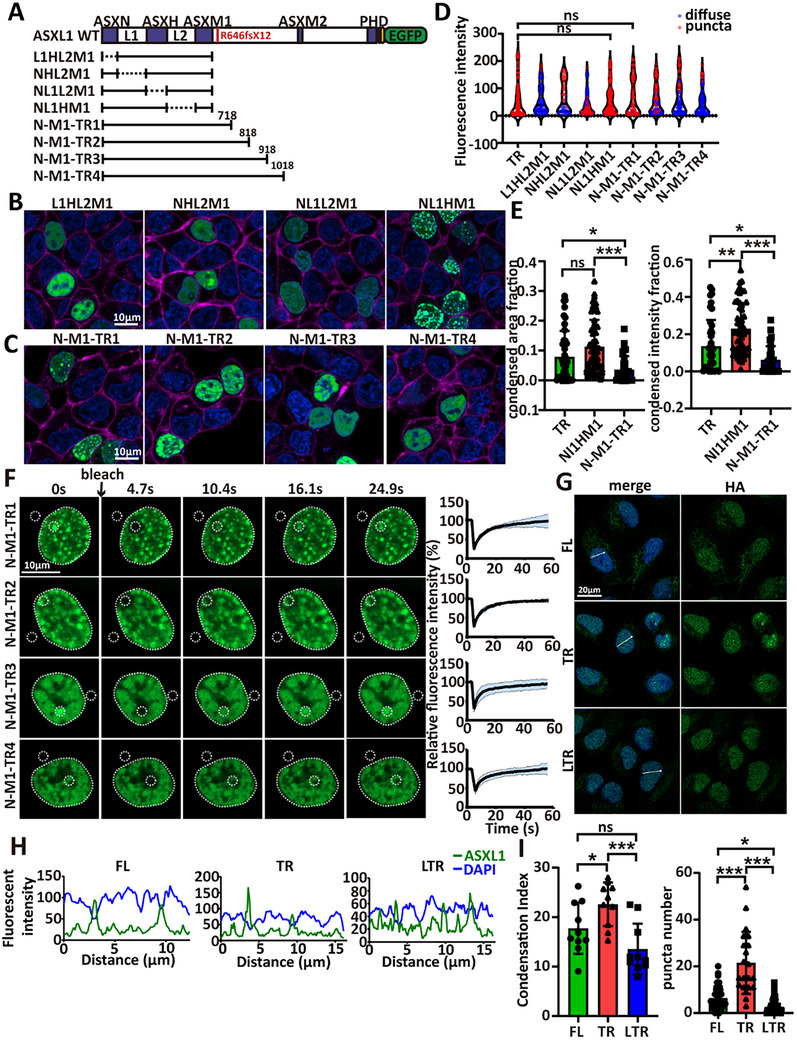
Only ASXL1 truncations within AA619–AA718 facilitate condensate formation. (A) Schematic of mEGFP‐tagged ASXL1 deletion mutants and N‐M1 truncation constructs. (B) Confocal images of mEGFP‐tagged ASXL1 deletion mutants in live 293T cells. (C) Confocal images of mEGFP‐tagged N‐M1‐TRs in live 293T cells. (D) Violin plot quantifying condensate formation for each deletion mutant and N‐M1 truncation, quantified using the same approach as for the N‐T constructs in Figure 2 (TR, *n* = 115; L1HL2M1, *n* = 72; NHL2M1, *n* = 48; NL1L2M1, *n* = 102; NL1HM1, *n* = 136; N‐M1‐TR1, *n* = 82; N‐M1‐TR2, *n* = 92; N‐M1‐TR3, *n* = 82; N‐M1‐TR4, *n* = 113). When each truncation was compared with ASXL1‐TR, p‐values were as follows: L1HL2M1 < 2.2 × 10^−16^; NHL2M1 = 4.011 × 10^−5^; NL1L2M1 = 3.339 × 10^−12^; NL1HM1 = 1; N‐M1‐TR1 = 1; N‐M1‐TR2 = 2.192 × 10^−10^; N‐M1‐TR3 < 2.2 × 10^−16^; N‐M1‐TR4 < 2.2 × 10^−16^. (E) Condensed burden quantified as condensed area fraction (left) and condensed intensity fraction (right) per nucleus from confocal images (expression‐matched; see Experimental Section) (TR, n = 40; NL1HM1, *n* = 52; N‐M1‐TR1, *n* = 29). Statistical analysis was performed using one‐way ANOVA with post‐hoc testing (^*^ *p* < 0.05, ^**^ *p* < 0.01, ^***^ *p* < 0.001; mean ± SD.) (F) Representative FRAP images (left) and corresponding fluorescence recovery curves (right) of N‐M1‐TRs. White dotted circles denote the bleached and background regions; the dashed line outlines the total fluorescence area. *n* = 5 biological replicates. (G) Immunofluorescence for ASXL1 in endogenous knock‐in cells (ASXL1‐FL, ASXL1‐TR, ASXL1‐LTR). (H) Line‐scan quantifications for ASXL1‐FL, ASXL1‐TR, ASXL1‐LTR. (I) Left: Condensation Index (CI) from nuclear line‐scans (see Experimental Section) quantifies puncta prominence (FL, *n* = 10; TR, *n* = 10; LTR, *n* = 10). Statistics: one‐way ANOVA with post‐hoc testing (^*^ *p* < 0.05, ^**^ *p* < 0.01, ^***^ *p* < 0.001; mean ± SD). Right: Number of puncta per cell (FL, TR, LTR). (FL, *n* = 31; TR, *n* = 21; LTR, *n* = 39). Statistics: one‐way ANOVA with post‐hoc testing (^*^ *p* < 0.05, ^**^ *p* < 0.01, ^***^ *p* < 0.001; error bars: mean ± SD).

To further define the domains required for condensate formation, we generated deletion mutants lacking individual regions: ASXN, L1 (ASXN‐ASXH linker), ASXH, L2 (ASXH‐ASXM linker), or ASXM1 (Figure 3A). Deletion of ASXN, L1, ASXH, or ASXM1 (N‐T5) each abolished condensate formation (Figures 2B–C and 3B,D), confirming that all four regions are necessary. In contrast, deletion of L2 had no negative effect (Figure 3B,D). In fact, the NL1HM1 mutant (lacking L2) showed higher per‐cell condensed burden than ASXL1‐TR (Figure 3E and Figure S3C) and retained liquid‐like properties (Figure S3D), indicating that L2 suppresses LLPS.

We next hypothesized that the region downstream of ASXM1, particularly the portion beyond AA646, inhibits condensate assembly. To pinpoint the specific region responsible for this inhibition, we constructed a panel of mutants (N‐M1‐TR1 to TR4) by progressively extending the truncation site 100–400 amino acids downstream of ASXM1 (Figure 3A). Only N‐M1‐TR1 (ending at AA718) retained a condensate‐forming capacity comparable to ASXL1‐TR, whereas longer variants (TR2‐TR4) showed progressively diminished condensation and increasingly irregular morphology (Figure 3C,D). All retained FRAP recovery (Figure 3F), indicating that they were not solid aggregates. These data identify a specific segment (AA719‐918) within the ASXL1 llIDR that impedes condensate formation.

To test this in an endogenous context, we generated HA‐tagged U2OS knock‐ins expressing ASXL1‐FL‐HA, ASXL1‐TR‐HA, and a long truncation (ASXL1‐LTR, which includes AA719‐918, formerly N‐M1‐TR3) (Figure S3E‐G) . Immunofluorescence detects discrete nuclear foci for ASXL1‐TR but not for ASXL1‐LTR (Figure 3G). Condensation Index (CI) and puncta number follow the same order (TR > FL > LTR; Figure 3H,I). These results further support a condensation inhibitory role for AA719‐918.

Notably, N‐M1‐TR1 showed a lower condensed area/intensity fraction than ASXL1‐TR (Figure 3E). At moderate expression, N‐M1‐TR1 also formed fewer condensates than TR, but this difference was not evident at higher expression (Figure S3C). Together, these findings suggest that AA646‐718 modestly dampen ASXL1's condensate‐forming potential.

Collectively, these data identify AA619–718 as a condensation‐promoting segment. This region closely overlaps with the mutation hotspot in myeloid malignancies (AA591–693), with the most common mutation at AA646 showing the strongest condensate phenotype. Truncations just upstream (e.g., N‐T5) or downstream (e.g., N‐M1‐TR1) showed attenuated condensation, suggesting that misregulated LLPS contributes to ASXL1‐driven pathogenesis.

### 2.3 Electrostatic Autoregulation of ASXL1 Condensates via a Cis Acidic–Basic Interaction

We identified a specific segment within ASXL1 llIDR (AA719‐918), hereafter termed the LLPS‐inhibiting region, that impedes condensate formation (Figure 3). To investigate the underlying mechanism, we analyzed the “IDR grammar” of this region—referring to the non‐random sequence patterns and compositional biases that influence phase separation and IDR behavior [24, 25, 26, 27].

**FIGURE 4:**
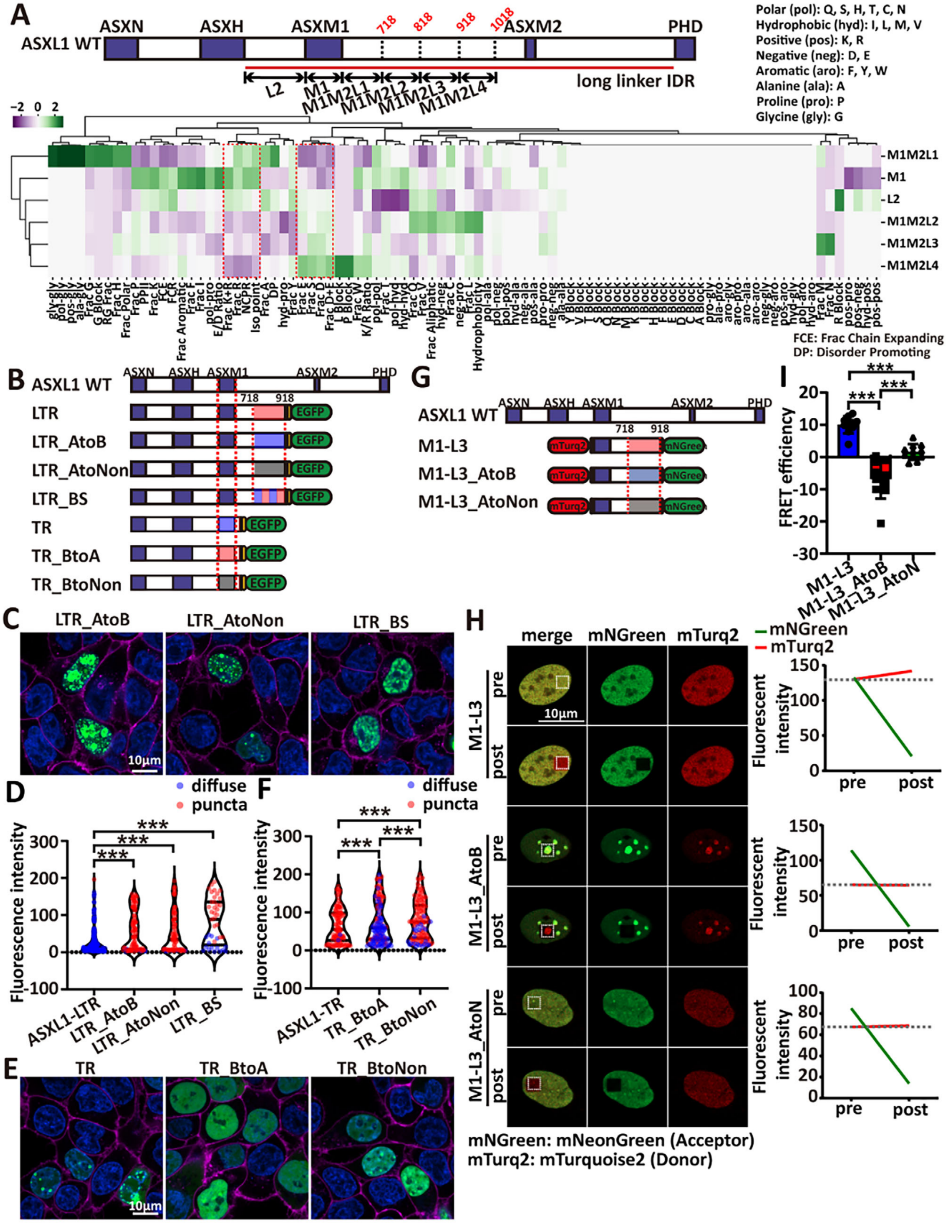
Cis charge pairing within ASXL1's llIDR provides an autoregulatory brake. (A) Clustering analysis of non‐random sequence features across ASXL1‐specific IDRs. Z‐scores for enriched (“blocky”) or depleted (“well‐mixed”) features are depicted on a green‐to‐purple color scale. Features groups: **Patterning** (36), NARDINI binary patterning z‐scores (blocky vs well‐mixed); **Amino‐acid fractions** (20), per‐residue composition; **Grouped composition** (5), K+R, D+E, Polar, Aliphatic, Aromatic; **Basic/acidic ratios** (2), log10(K/R), log10(E/D); **Charge/global** (7), FCR (fraction charged residues), NCPR (net charge per residue), hydrophobicity, disorder‐promoting, pI (isoelectric point), chain‐expanding, PPII (Polyproline II Propensity); **Residue blocks** (19), run/patch fractions; **RG‐stretch** (1), RG‐rich run fraction. (B) Schematic of mEGFP‐tagged ASXL1‐LTR and ASXL‐TR mutant constructs. (C) Confocal images of mEGFP‐tagged LTR mutants in live 293T cells. (D) Violin plot quantifying condensate formation for each LTR‐mutant construct (ASXL1‐LTR, *n* = 131; LTR_AtoB, *n* = 97; LTR_AtoNon, *n* = 82; LTR_BS, *n* = 41). ASXL1‐LTR was used as a negative control and analyzed in the same manner as the N‐T constructs. Statistical comparisons versus ASXL1‐LTR yielded the following p‐values: LTR_AtoB < 2.2 × 10^−16^; LTR_AtoNon < 2.2 × 10^−16^; LTR_BS = 6.019 × 10^−7^. (E) Confocal images of mEGFP‐tagged TR mutants in live 293T cells. (F) Violin plot quantifying condensate formation for each TR‐mutant construct (TR, *n* = 110; TR_BtoA, *n* = 143; TR_BtoNon, *n* = 126). ASXL1‐TR was used as a negative control and analyzed in the same manner as the N‐T constructs. Statistical comparisons versus ASXL1‐TR yielded the following p‐values: TR_BtoA < 2.2 × 10^−16^; TR_BtoNon = 1.283 × 10^−5^. (G) Schematic of mTurquoise2– [M1–L3]–mNeonGreen and mTurquoise2– [M1–L2L3]–mNeonGreen cis FRET sensor and their charge‐disrupting variants (AtoB, acidic→basic, AtoNon, acidic→neutral). (H) Representative photobleach FRET images (left) and fluorescent intensity (mTurquoise2 and mNeonGreen) changes between pre‐bleach and after‐bleach of M1–L3, M1–L3_ AtoB, and M1–L3_ AtoNon. (I) FRET efficiency (see Experimental Section) for [M1–L3] sensors (M1‐L3, *n* = 10; M1‐L3_AtoB, *n* = 10; M1‐L3_AtoNon, *n* = 10). Statistics: one‐way ANOVA with post‐hoc testing (^*^ *p* < 0.05, ^**^ *p* < 0.01, ^***^ *p* < 0.001; error bars: mean ± SD).

Using a published feature‐analysis pipeline [25], we examined six IDR segments spanning the region from L2 to the truncation site of N‐M1‐TR4 (AA361‐1018): the L2 linker (AA361‐522); the LLPS‐promoting ASXM1 domain (AA523‐618); M1M2L1(AA619‐718); and three LLPS‐inhibiting segments—M1M2L2 (AA719‐818), M1M2L3 (AA819‐918), and M1M2L4 (AA919‐1018). Each was annotated using a 90‐feature vector capturing amino acid composition and non‐random binary sequence patterns [25]. This analysis revealed an opposing charge distribution: ASXM1 was enriched in basic (positively charged) residues, whereas M1M2L2 and M1M2L3 were enriched in acidic (negatively charged) residues (Figure 4A). We hypothesized that these acidic segments electrostatically interact with ASXM1, masking its condensation interface and preventing LLPS.

To test this, we introduced charge‐altering mutations into the M1M2L2 and M1M2L3 within the ASXL1‐LTR (N‐M1‐TR3) background. We generated three variants (Figure 4B): (1) LTR_AtoB, in which acidic residues were replaced with basic residues; (2) LTR_AtoNon, where acidic residues were replaced with neutral residues; and (3) LTR_BS, which featured partial acidic‐to‐basic substitutions to achieve charge balance. All three mutants significantly enhanced condensate formation compared to ASXL1‐LTR (Figure 4C,D), with condensed burden rising in a charge‐graded manner (LTR_BS < LTR_AtoNon < LTR_AtoB; Figure S4A). Notably, LTR_AtoB puncta displayed irregular morphology, minimal FRAP recovery (Figure S4B), and colocalized with nucleoli (Figure 4C), possibly due to enhanced electrostatic attraction to negatively charged nucleolar components [25]. In contrast, LTR_AtoNon and LTR_BS retained spherical morphology and fast fluorescence recovery (Figure S4B).

Conversely, charge‐reversals of ASXM1 in the TR background (TR_BtoA: basic→acidic; TR_BtoNon: basic→neutral, Figure 4B) reduced condensate formation (Figure 4E,F), with condensed burden decreasing (TR > TR_BtoNon > TR_BtoA, Figure S4C). Together, these results indicate electrostatics within ASXL1 tune condensation.

To probe electrostatics directly, we performed permeabilized‐cell assays in U2OS cells expressing ASXL1‐TR–EGFP (Figure S4D). Acute high salt (300 mm NaCl) dispersed ASXL1‐TR condensates and abolished DNA co‐localization, both of which recovered after washout (Figure S4E,F), consistent with electrostatic contributions to condensate integrity and DNA engagement.

We next asked whether an intramolecular charge interaction restrains condensation. Using an acceptor‐photobleach FRET cis sensor (mTurquoise2– [M1–L3]–mNeonGreen) (Figure 4G), WT M1–L3 showed robust FRET (∼10%), whereas charge‐disrupting variants AtoB (∼−6%) and AtoNon (∼2%) exhibited near‐baseline FRET signals (Figure 4H). Similar outcomes were obtained when M1M2L1 was replaced by a neutral linker (Figure S4G–I), supporting a specific intramolecular proximity between the basic ASXM1 segment and the acidic M1M2L2/L3 block. Together with the salt sensitivity, these data indicate that ASXL1‐TR condensate formation and DNA association are driven by electrostatic interactions, and that cis pairing between ASXM1 (basic) and M1M2L2/L3 (acidic) provides an autoregulatory brake on phase separation.

To assess whether similar regulatory logic exists in other proteins, we identified 101 llIDRs across 99 proteins in human proteome (>1000 residues; Table S1). These proteins were enriched for structural [28, 29] and chromatin‐regulatory functions (Figure S5A and Table S2). Notably, many pathogenic variants in these chromatin‐associated proteins mapped within llIDRs (Figure S5B), suggesting functional importance. Feature variability analysis showed charge‐related properties had the highest intra‐IDR diversity (Figure S5C), suggesting that electrostatic autoregulation may be a common mechanism in llIDR‐containing proteins.

### 2.4 ASXL1‐TR Condensate Formation is Associated With Neutrophil Differentiation

Given that ASXL1's condensation‐promoting region overlaps a major mutational hotspot in myeloid malignancies. Previous works showed that ASXL1 is essential for neutrophil differentiation [30, 31, 32, 33]. Therefore, we next asked whether condensate formation affects neutrophil differentiation. To evaluate this in a human cellular context, we used HL‐60 cells, which undergo neutrophil differentiation upon Tretinoin treatment [34].

**FIGURE 5:**
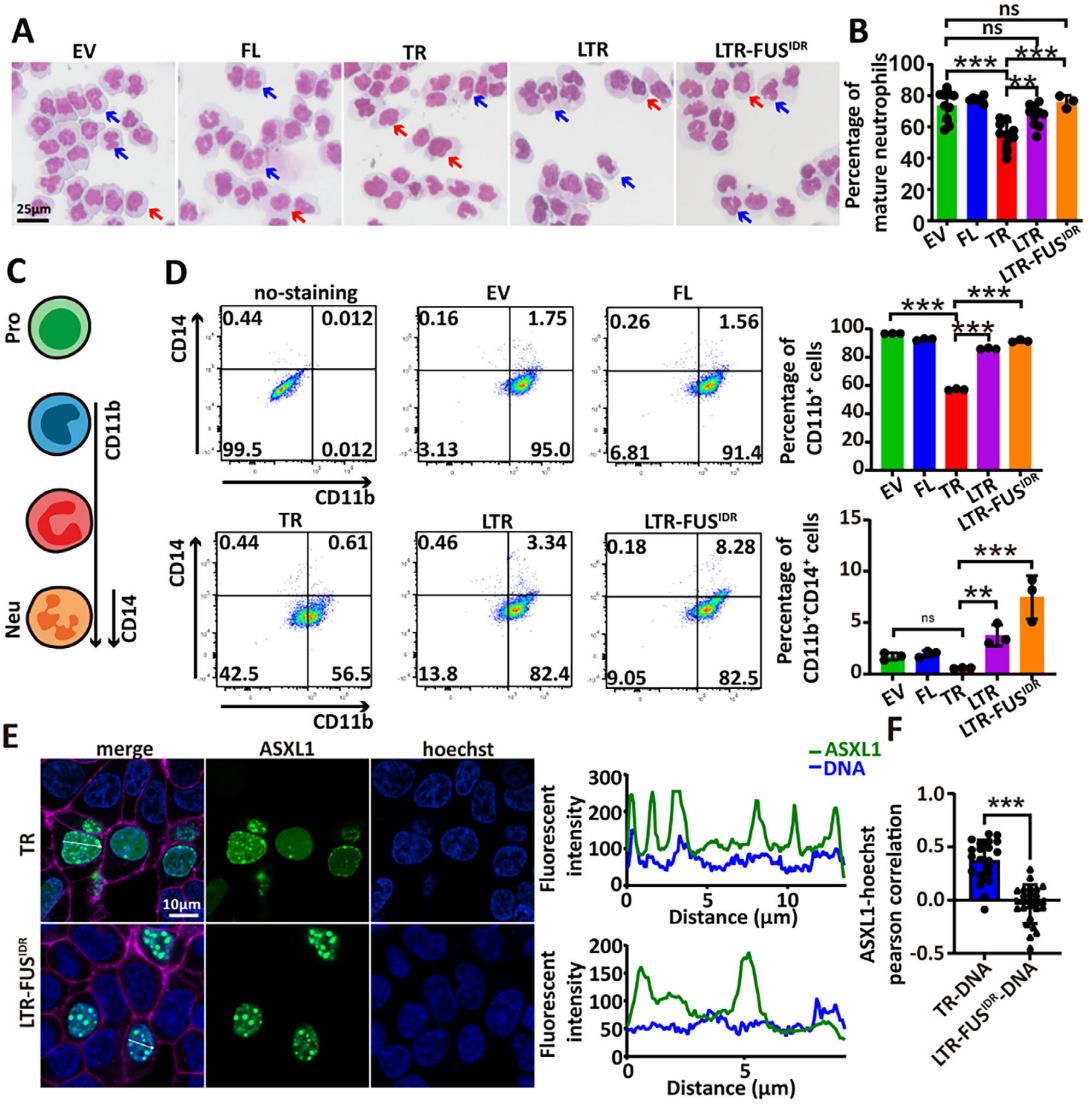
ASXL1‐TR condensate formation inhibits neutrophil maturation. (A) May‐Grünwald‐Giemsa staining of HL‐60 cells differentiate with Tretinoin for 5 days. (scale bar: 25 µm). Blue arrows indicate mature neutrophils; red arrows indicate immature neutrophils (B) Quantification of neutrophils differentiation based on nuclear morphology. Statistical analysis was performed using one‐way ANOVA followed by Fisher's LSD post hoc test (^*^ *p* < 0.05, ^**^ *p* < 0.01, ^***^ *p* < 0.001; error bars, mean ± SD) (C) Schematic overview of surface marker expression during neutrophil differentiation. (D) Flow cytometry analysis of CD11b^+^ neutrophils and CD11b^+^CD14^low^ mature neutrophils. Statistical analysis was performed using one‐way ANOVA followed by Fisher's LSD post hoc test (^*^ *p* < 0.05, ^**^ *p* < 0.01, ^***^ *p* < 0.001; error bars, mean ± SD). (E) Representative images and line‐scan quantifications for ASXL1‐TR‐mEGFP and ASXL1‐LTR‐FUS^IDR^‐mEGFP. (F) Pearson's r for TR ↔ Hoechst and LTR‐FUS^IDR^ ↔ Hoechst. Statistics: two‐tailed Student's *t*‐test (^*^ *p* < 0.05, ^**^ *p* < 0.01, ^***^ *p* < 0.001; mean ± SD).

We generated stable HL‐60 lines expressing empty vector (EV), ASXL1‐FL, the condensate‐forming truncation ASXL1‐TR, and the non‐condensating truncation ASXL1‐LTR. Lentiviral integration and protein expression were confirmed by PCR and Western blotting, respectively (Figure S6A,B). ASXL1‐FL protein was difficult to detect, likely due to reduced stability [9]. Morphologically, after Tretinoin treatment, ASXL1‐TR significantly reduced the proportion of mature neutrophils with segmented nuclei compared to EV and ASXL1‐FL, while ASXL1‐LTR had a minimal effect and did not differ significantly from EV (Figure 5A,B).

To complement the morphological readouts, we profiled CD11b and CD14 by flow cytometry. CD11b is upregulated early during neutrophil maturation [35], whereas CD14—primarily a monocyte marker—can be present at low levels in terminally differentiated neutrophils [36, 37, 38]. We therefore used CD11b as the primary flow cytometric readout and interpreted CD14 conservatively. ASXL1‐TR expression reduced the frequency of CD11b^+^ cells relative to ASXL1‐LTR, consistent with an impaired maturation trajectory; the CD11b^+^CD14^low^ compartment showed the same direction of change but with lower dynamic range (Figure 5C–D). Together with the nuclear‐segmentation data, these results indicate that condensate‐forming ASXL1‐TR compromises neutrophil maturation, whereas the non‐condensing ASXL1‐LTR does not.

To test whether condensate formation alone is sufficient to block differentiation, we fused ASXL1‐LTR with FUS^IDR^, a well‐characterized phase separation domain [39], generating ASXL1‐LTR‐FUS^IDR^. This fusion protein formed robust nuclear condensates in 293T cells (Figure S6C–E), confirming successful LLPS induction. However, unlike ASXL1‐TR, ASXL1‐LTR‐FUS^IDR^ did not impair HL‐60 differentiation. In fact, the proportion of CD11b^+^CD14^low^ mature neutrophils was even higher than in EV cells (Figure 5D), and May–Grünwald–Giemsa staining showed no difference in the proportion of mature neutrophils with segmented nuclei (Figure 5A,B).

Live‐cell imaging and localization analysis showed that ASXL1‐LTR‐FUS^IDR^ condensates exhibited minimal overlap with Hoechst‐stained DNA, in sharp contrast to the DNA‐associated ASXL1‐TR condensates (Figure 5E,F). Additionally, the molecular weight of ASXL1‐LTR‐FUS^IDR^ matched its predicted size, whereas ASXL1‐TR and ASXL1‐LTR appeared larger, possibly due to post‐translational modification (Figure S6B). This suggests that the FUS^IDR^ fusion may alter ASXL1's biochemical state or localization. Together, these findings indicate that LLPS competence is necessary but not sufficient—electrostatic (charge‐encoded) targeting to DNA‐rich chromatin is also required for ASXL1‐TR condensates to disrupt neutrophil differentiation.

### 2.5 BRD2 is Incorporated Into ASXL1‐TR Condensates

Having established that ASXL1‐TR condensate formation correlates with disrupted neutrophil differentiation, we next sought to determine what molecular processes occur within these condensates. ASXL1 functions as a chromatin scaffold that coordinates various epigenetic regulators [15]. We hypothesized that ASXL1‐TR condensation may reorganize cofactor recruitment, thereby influencing chromatin regulation. To identify condensate‐resident proteins, we employed BioID2 proximity labeling followed by mass spectrometry (BioID2‐MS) [40], enabling an unbiased screen for proteins enriched near ASXL1‐TR.

**FIGURE 6:**
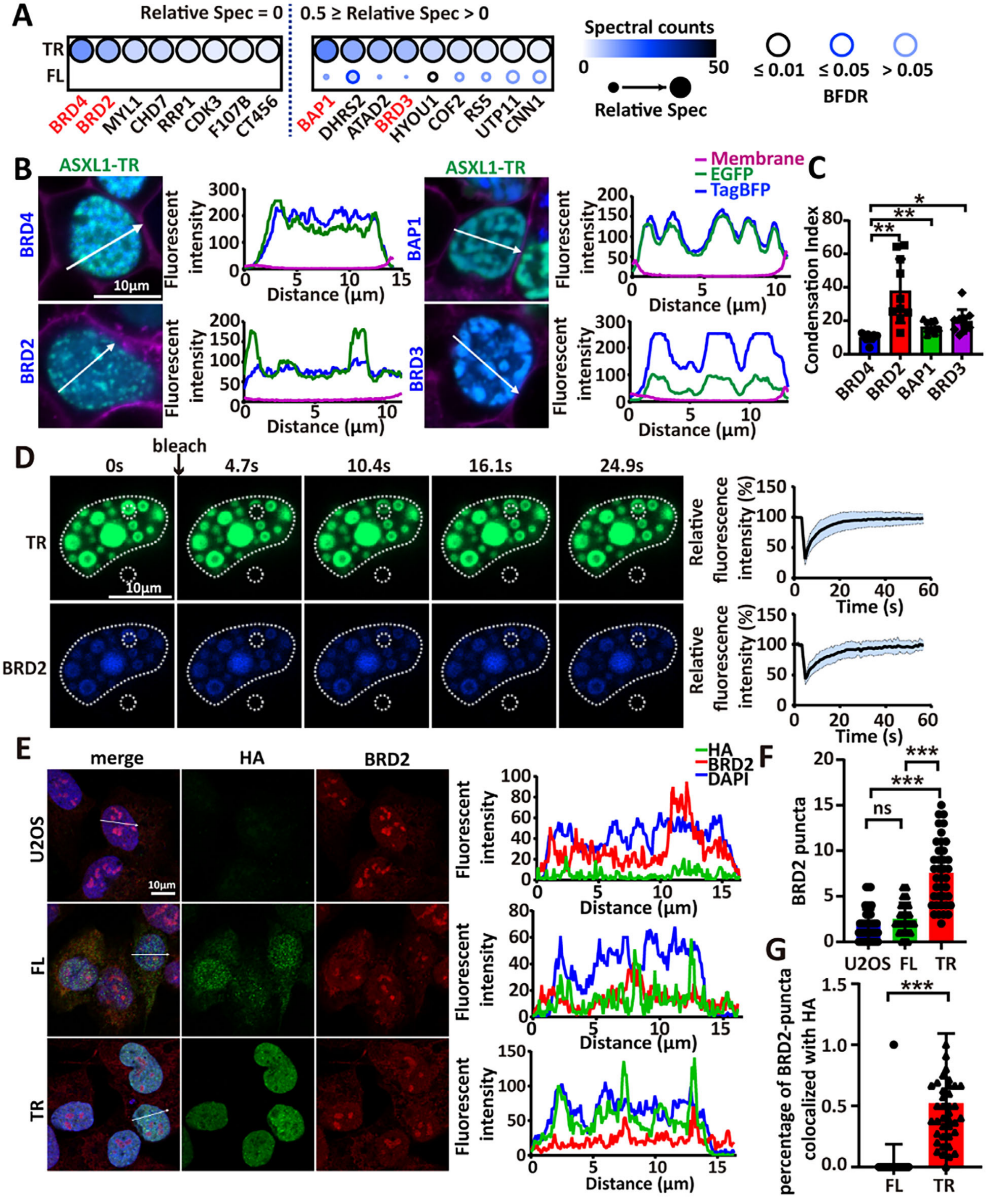
BRD2 incorporates into ASXL1‐TR condensates. (A) Dot plot of candidate cofactors enriched in ASXL1‐TR proximity, visualized using ProHits‐viz platform [45]. (Relative spec ≤ 0.5 in ASXL1‐FL) (B) Representative confocal images and line‐scan quantifications of ASXL1‐TR‐mEGFP co‐expressed with BRD4‐, BRD2‐, BAP1‐, or BRD3‐TagBFP. (C) Condensation Index (CI) from nuclear line‐scans (see Experimental Section) quantifies puncta prominence (BRD4, *n* = 10; BRD2, *n* = 10; BAP1, *n* = 10; BRD3, *n* = 10). Statistics: one‐way ANOVA with post‐hoc testing (^*^ *p* < 0.05, ^**^ *p* < 0.01, ^***^ *p* < 0.001; mean ± SD). (D) Representative FRAP images (left) and corresponding fluorescence recovery curves (right) in cells co‐expressing ASXL1‐TR and BRD2. White dotted circles indicate the bleached region and background; the dashed line outlines the total fluorescence region. *n* = 5 biological replicates. (E) Representative immunofluorescence images (left) and line‐scan quantifications (right) showing subnuclear distribution of BRD2 in U2OS cells expressing HA‐tagged ASXL1‐FL and ASXL1‐TR. (F) Quantification of BRD2 puncta per cell in wild‐type U2OS, ASXL1‐FL, and ASXL1‐TR cells. Statistical analysis: one‐way ANOVA with Fisher's LSD post hoc test (^*^ *p* < 0.05, ^**^ *p* < 0.01, ^***^ *p* < 0.001; error bars, mean ± SD). (G) Percentage of BRD2 puncta colocalizing with ASXL1‐HA. Statistical analysis: two‐tailed Student's *t*‐test (^*^ *p* < 0.05, ^**^ *p* < 0.01, ^***^ *p* < 0.001; error bars, mean ± SD).

We generated U2OS cell lines stably expressing BioID2‐tagged ASXL1‐FL, ASXL1‐TR, or empty vector (Figure S7A). U2OS cells were chosen for their flat morphology, which facilitates imaging, and because ASXL1 is broadly expressed, increasing generalizability of findings. Western blotting confirmed robust fusion protein expression (Figure S7B), and immunofluorescence showed that ASXL1‐TR‐BioID2 retained its condensate‐forming ability (Figure S7C). Upon biotin treatment, proximal proteins were labeled, enriched via streptavidin pulldown, and identified by MS (Table S3). To focused on candidate‐specific factors, we prioritized proteins with a relative spectral count ≤ 0.5 in the ASXL1‐FL dataset (Figure 6A and Table S4).

To prioritize validation, we selected nuclear‐localized candidates among the enriched proteins [41]. This yielded 10 candidates: BRD2, BRD3, BRD4, BAP1, CHD7, CT45A6, ATAD2, RRP1, UTP11, and F107B. TagBFP‐tagged constructs were generated for all except CHD7 and F107B (due to cDNA amplification failure). Co‐expression with ASXL1‐TR‐mEGFP revealed that BAP1, BRD2, BRD3, and BRD4 colocalized with ASXL1‐TR condensates, while the remaining candidates did not (Figure 6B and Figure S7D,E).

Among the validated hits, BAP1 and BRD4 are known ASXL1 interactors [42, 43], supporting the screen's specificity. Condensates index (CI) analyses indicated that BRD4 reduces ASXL1‐TR condensate burden (Figure 6B), so we prioritized BRD2, BAP1, and BRD3 for follow‐up. To assess which cofactors were stably integrated into ASXL1‐TR assemblies, we examined morphology and FRAP. Co‐expression with BRD3 or BAP1 produced irregular condensates with slower recovery (>40 s), whereas BRD2 co‐expression preserved spherical morphology and fast recovery (<20 s), comparable to ASXL1‐TR alone (Figure 6C and Figure S7F). These observations suggest BRD2 is the most compatible cofactor with LLPS‐like ASXL1‐TR condensates.

To test whether BRD2 recruitment depends on ASXL1‐TR condensation, we examined endogenous BRD2 in wild‐type and ASXL1‐FL/ASXL1‐TR U2OS cells. In EV and ASXL1‐FL cells, BRD2 appeared nucleolar or diffuse with few puncta, whereas ASXL1‐TR–expressing cells showed increased BRD2 puncta that largely colocalized with ASXL1‐TR condensates (Figure 6D–F). In co‐expression assays, BRD2 formed condensates with ASXL1‐TR but not with ASXL1‐LTR (Figure S8A,B, indicating that condensate formation is required for BRD2 recruitment under our conditions. Moreover, BRD2 was recruited by NL1HM1 but not NL1H (Figure S8C,D), consistent with dependence on the ASXM1 domain, which harbors a known BRD‐binding motif [44]. Collectively, these data support a model in which BRD2 is recruited to ASXL1‐TR condensates via ASXM1‐mediated interaction.

### 2.6 ASXL1‐TR Condensates Redirect BRD2 to Ectopic Genomic Localization

**FIGURE 7:**
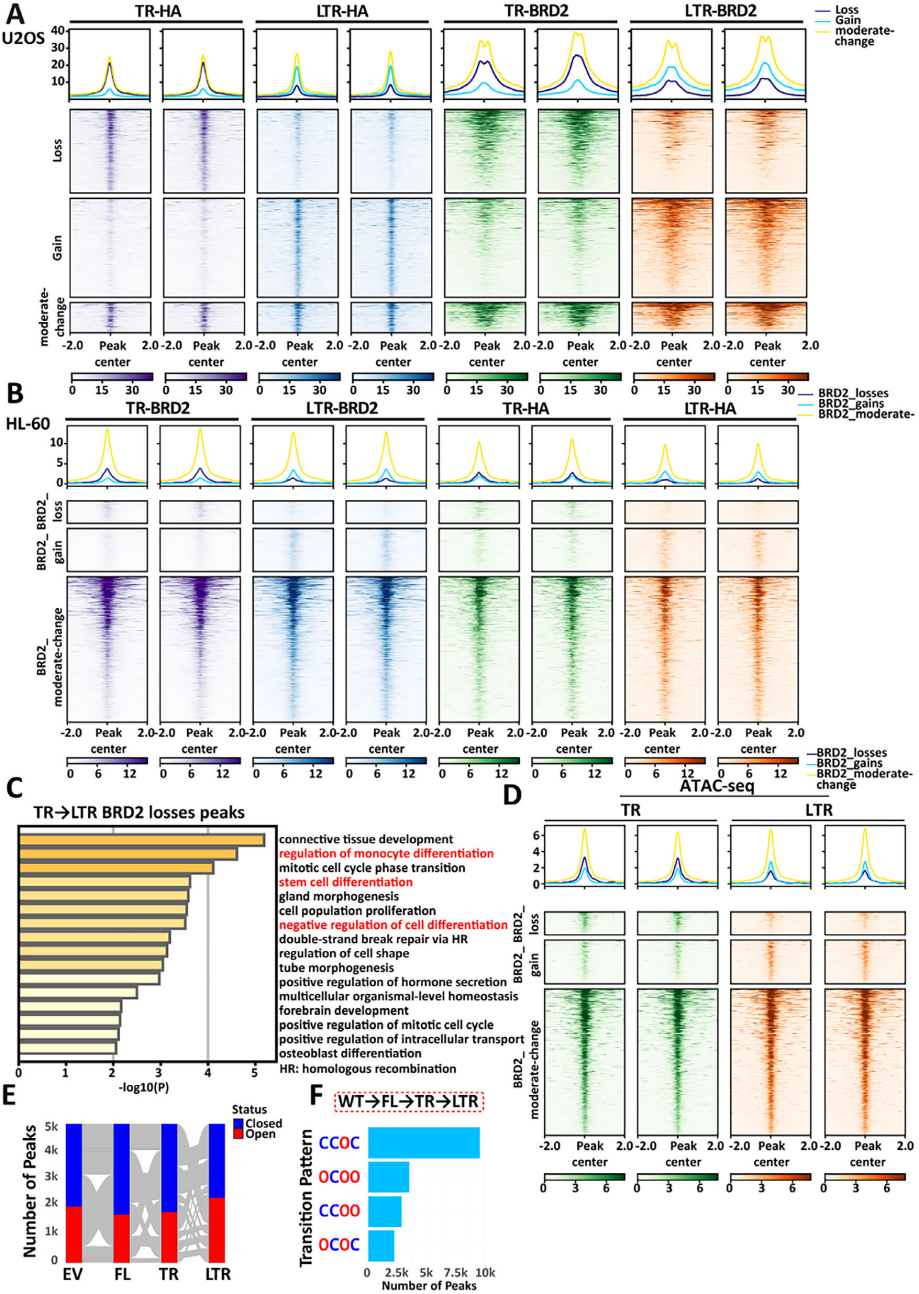
ASXL1‐TR condensates alter BRD2 genomic localization. (A) ChIP‐seq profiles of ASXL1‐TR‐HA, ASXL1‐LTR‐HA, and BRD2 at ASXL1‐differential peaks (TR vs LTR) in U2OS. (B) CUT&Tag profiles of BRD2 and HA‐tagged ASXL1 at BRD2‐differential peaks (TR vs LTR) in HL‐60. (C) Gene ontology (GO) enrichment of genes annotated to BRD2‐loss (TR→LTR) peaks (cell differentiation‐related terms highlighted). (D) ATAC‐seq chromatin accessibility at BRD2‐differential peaks in HL‐60 cells expressing ASXL1‐TR or ASXL1‐LTR. (E) Alluvial plot showing representative chromatin‐accessibility transitions across HL‐60 lines expressing empty vector (EV), ASXL1‐FL, ASXL1‐TR, and ASXL1‐LTR. A random subset of 5000 peaks is shown for clarity. Each flow represents peaks with the same four‐state pattern (Open = red; Closed = blue). (F) Bar plot of transition patterns among peaks that became accessible specifically in ASXL1‐TR (i.e., FL→TR, Closed→Open). Patterns are encoded as four‐letter codes (C = Closed, O = Open) corresponding to state order EV→FL→TR→LTR.

C‐terminal truncation of ASXL1 (ASXL1‐TR) induces DNA‐associated condensate formation and recruits BRD2, which is otherwise diffusely localized. To assess whether this recruitment alters BRD2's genomic binding, we performed ChIP‐seq for HA‐tagged ASXL1 and BRD2 in U2OS cells expressing either ASXL1‐TR or ASXL1‐LTR. Differential ASXL1‐HA peaks were categorized into loss (reduced in LTR; log_2_FC < –1), gain (increased in LTR; log_2_FC > 1), or moderate‐change (–1 ≤ log_2_FC ≤ 1) groups based on log2 fold change and statistical significance (FDR < 0.05). BRD2 occupancy shifted in parallel with ASXL1 redistribution across these regions (Figure 7A). In HL‐60 cells expressing ASXL1‐TR or ASXL1‐LTR, CUT&Tag showed that BRD2‐differential peaks displayed parallel changes in ASXL1‐HA occupancy (co‐enrichment) (Figure 7B), supporting the notion that ASXL1‐TR condensates redirect BRD2 to ectopic chromatin loci in a myeloid context. Gene ontology (GO) analysis of genes annotated to BRD2‐loss (TR→LTR) peaks was enriched for terms related to monocyte/stem‐cell differentiation and negative regulation of differentiation (Figure 7C) and included loci such as *HOXA7* (Figure S9A), a member of *HOXA* cluster that is highly expressed in AML patients [46, 47]. These data are consistent with lineage‐program perturbation.

Previous studies have shown that BRD2‐containing condensates can promote accessible chromatin compartmentalization in the absence of cohesin [48, 49]. To determine whether BRD2 relocalization affects chromatin state, we performed ATAC‐seq in HL‐60 cells expressing either ASXL1‐TR or ASXL1‐LTR. At BRD2‐differential peaks, ATAC‐seq signal positively tracked BRD2 occupancy (Figure 7D), consistent with BRD2's known association with accessible chromatin.

We profiled ATAC‐seq across HL‐60 lines expressing empty vector (EV), ASXL1‐FL, ASXL1‐TR, and ASXL1‐LTR. A unified reference peak set was generated, and a binary open/closed state was assigned for each condition. Alluvial plots of 5,000 randomly selected peaks revealed shifts in accessibility, most notably from ASXL1‐TR to ASXL1‐LTR (Figure 7E). Of the 18,203 peaks uniquely opened in ASXL1‐TR (i.e., closed in FL and open in TR), only ∼35% remained accessible in ASXL1‐LTR, whereas ∼65% reverted to closed (Figure 7F). These findings support a model in which ASXL1‐TR condensates promote chromatin accessibility that is reversed when LLPS is blocked.

Similar to the HL‐60 lines, we also performed ATAC‐seq in ASXL1‐TR and ASXL1‐LTR knock‐in U2OS cells. At BRD2‐differential peaks, ATAC‐seq signal tracked BRD2 occupancy (Figure S9B), and genome‐wide transition trends mirrored HL‐60 (Figure S9C,D). Together with the HL‐60 results, these data indicate that ASXL1‐TR expression is associated with increased accessibility at a subset of genomic sites, and that this accessibility is largely reversed by ASXL1‐LTR. Because neutrophil maturation involves extensive genome condensation (“supercontraction”) [50], such mis‐targeted accessibility may contribute to impaired differentiation.

In sum, ASXL1‐TR is associated with BRD2 relocalization to ectopic sites, accompanied by mis‐targeted accessibility; these changes may contribute to impaired neutrophil differentiation.

### 2.7 Tosedostat Reduces ASXL1‐TR Condensate Burden and Partially Improves Nuclear Segmentation

Our findings suggest that ASXL1‐TR condensate formation may impair neutrophil maturation (Figure 5) and potentially underlie myeloid malignancy progression [30]. To explore therapeutic potential, we screened for compounds that reduce ASXL1‐TR condensates and tested whether this could restore differentiation.

**FIGURE 8:**
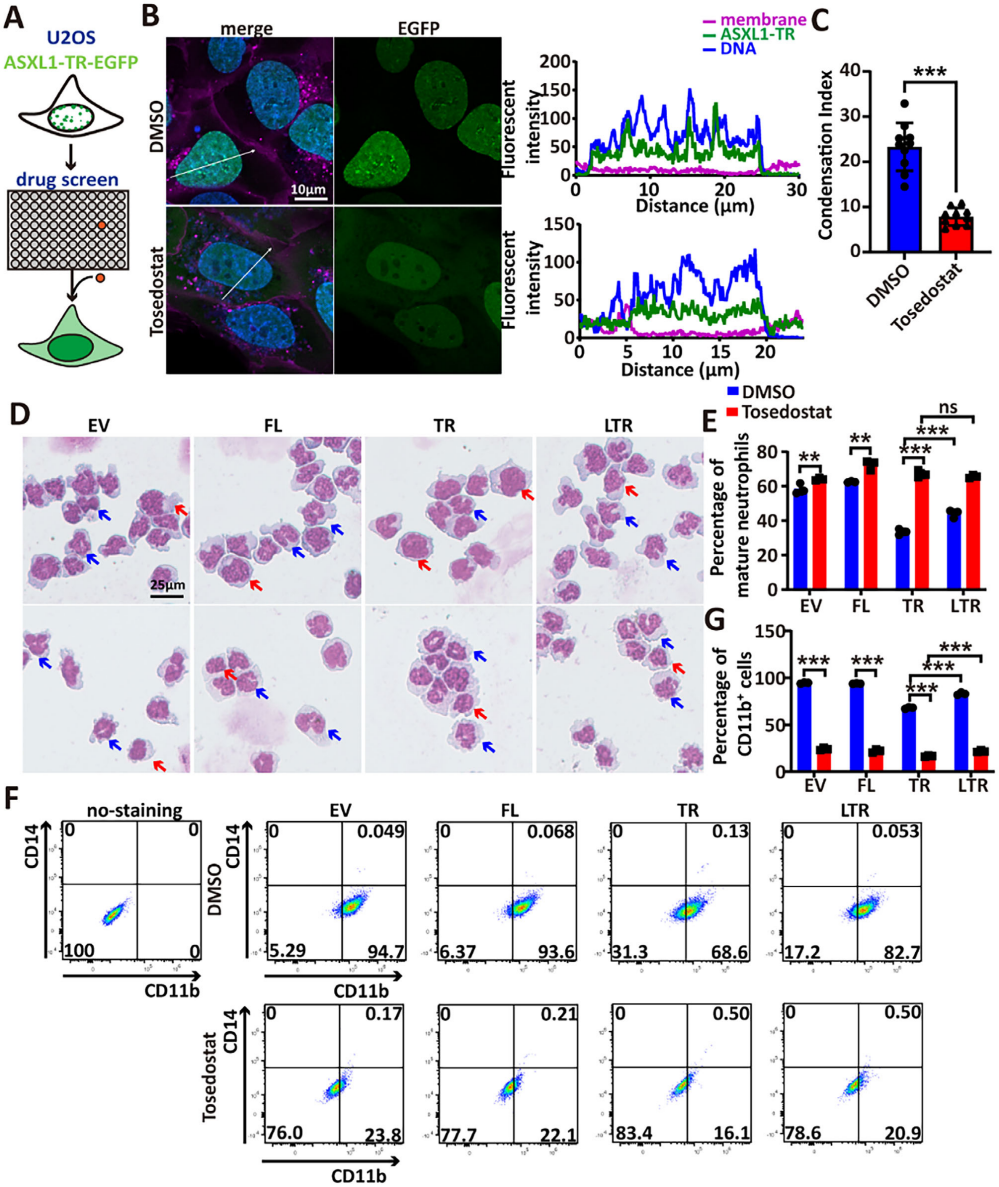
Tosedostat increases neutrophil nuclear segmentation in HL‐60. (A) Schematic of the small‐molecule screening workflow used to identify compounds that reduce ASXL1‐TR condensate formation. (B) Confocal images (left) and line‐scan quantifications (right) of mEGFP‐tagged ASXL1‐TR in a stable U2OS cell line following 24 h treatment with DMSO or Tosedostat. (C) Condensation Index (CI) from nuclear line‐scans (see Experimental Section) quantifies puncta prominence (DMSO, *n* = 10; Tosedostat, *n* = 10. Statistics: two‐tailed Student's *t*‐test (^*^ *p* < 0.05, ^**^ *p* < 0.01, ^***^ *p* < 0.001; mean ± SD) (D) May‐Grünwald‐Giemsa staining of HL‐60 cells differentiated with DMSO + Tretinoin or Tosedostat + Tretinoin. Blue arrows, mature neutrophils; red arrows, immature neutrophils. (E) Quantification of segmented neutrophils based on nuclear morphology. Statistics: one‐way ANOVA with post‐hoc testing. (^*^ *p* < 0.05, ^**^ *p* < 0.01, ^***^ *p* < 0.001; mean ± SD) (F) Flow‐cytometry analysis of CD11b^+^ and CD14^+^ surface markers ± tosedostat after tretinoin induction. (G) Quantification of CD11b^+^ cells across groups. Statistics: one‐way ANOVA with post‐hoc testing. (^*^ *p* < 0.05, ^**^ *p* < 0.01, ^***^ *p* < 0.001; mean ± SD).

We established a U2OS cell line stably expressing ASXL1‐TR‐mEGFP, in which condensates were readily detectable by live confocal imaging (Figure 8A). Screening 160 compounds (Table S5 and Figure S10), we identified the aminopeptidase inhibitor Tosedostat as a hit that significantly reduced ASXL1‐TR condensation (Figure 8B,C).

To assess whether Tosedostat can rescue the differentiation block caused by ASXL1‐TR, we used HL‐60 cells stably expressing ASXL1‐FL, ASXL1‐TR, ASXL1‐LTR, or an empty vector control. Cells were treated with Tretinoin to induce neutrophil differentiation, in the presence or absence of Tosedostat. May–Grünwald–Giemsa staining revealed that Tosedostat treatment increased the proportion of segmented neutrophils across all groups, eliminating the previously observed difference between ASXL1‐TR and control cells (Figure 8D,E). However, flow cytometry revealed that CD11b^+^ cells frequency decreased in all groups after Tosedostat treatment, relative to DMSO control (Figure 8F,G). Importantly, the CD11b^+^ fraction in ASXL1‐TR cells remained lower than in EV/FL/LTR despite improved morphology (Figure 8F,G).

Together, tosedostat reduced ASXL1‐TR condensate burden and was associated with improved nuclear segmentation, whereas surface marker expression was not fully normalized—suggesting these maturation features may be partly independent.

## 3 Discussion

Intrinsically disordered regions (IDRs) are increasingly recognized as programmable regulators of condensation and nuclear organization, yet how their sequence encodes context‐specific control remains incompletely understood. Here, we define a long IDR within ASXL1 that functions as a self‐contained, charge‐based autoregulatory module. An upstream basic, condensation‐prone platform (centered on ASXM1) is restrained in cis by a downstream acidic “charge block.” Truncations that remove this inhibitory segment expose the basic platform, shift ASXL1 into a condensation‐competent state, redirect BRD2 to atypical genomic sites, and impair neutrophil maturation.

This framework explains graded outcomes across truncation sites [51]. Variants that fully remove the acidic block show strong condensation and differentiation defects, whereas those retaining portions of the acidic region display intermediate behavior. These transitions align with the clinical mutation hotspot (aa 591–693), suggesting that deregulated platform exposure contributes to leukemogenic risk. Charge‐rewiring experiments further demonstrate sufficiency: acidic→basic/neutral substitutions rescue condensation in an LTR background, while basic→acidic/neutral substitutions attenuate condensation in TR, supporting a model in which the ASXL1 llIDR toggles between repressed and condensation‐competent states via charge balance. In wild‐type ASXL1, dynamic regulation may involve downstream domains (e.g., the PHD finger [52]) or cofactors that modulate this cis brake.

In both U2OS knock‐in and hematopoietic models, ASXL1‐TR condensates recruit BRD2, and BRD2 occupancy tracks changes in chromatin accessibility at selected genomic sites; these site‐restricted shifts are consistent with a mechanism of cofactor retargeting rather than a global change in chromatin state. The *HOXA7* locus in HL‐60 exemplifies disease‐relevant targeting. Thus, BRD2 mislocalization by the exposed ASXL1 platform offers a parsimonious link between truncation, chromatin engagement, and maturation defects.

Notably, ASXL1‐TR condensates often display rim‐enriched (“ring‐like”) intensity profiles, sometimes persisting with BRD2 co‐enrichment. Optical sectioning can contribute (equatorial planes through 3D droplets yield annular profiles), but the persistence of brighter rims is also consistent with interfacial partitioning in multi‐component, electrostatically driven condensates [53], in which basic modules preferentially enrich at polyanion‐rich boundaries. A full dissection of core–shell composition is beyond scope, but ASXL1 provides a tractable in vivo system for future studies of interface‐biased architectures.

A previous report concluded that ASXL1 truncation reduces condensation [20]. We find that this discrepancy likely reflects experimental configuration (notably N‐terminal tagging), cell context, and expression level. In our hands, N‐terminal tags in HeLa reduced condensate formation and DNA association (Figure S1C,D), and tag identity also modulated behavior (Figure S1B). To minimize artifacts, we report tag orientation explicitly, validate with a short HA tag, and base key conclusions on endogenous knock‐in and myeloid‐cell assays. We also observe that ASXL1‐FL retains an intrinsic self‐association propensity that is normally restrained; under permissive conditions, FL can form condensates, consistent with autoinhibition. Together, these data motivate tag‐aware and, where possible, tag‐free endogenous readouts.

Our conclusions align with Latacz et al. [54], who similarly infer that removal of a negatively charged region promotes phase separation. We extend this by mapping the cis inhibitory subsegment, demonstrating bidirectional control via charge rewiring, and linking the exposed platform to BRD2 retargeting and maturation defects. Finally, a small‐molecule screen identified tosedostat as an example compound that reduces ASXL1‐TR condensate burden and is associated with partial restoration of nuclear segmentation; given its broad aminopeptidase activity, we interpret this as indirect and present our ASXL1‐TR–mEGFP U2OS line as a tractable platform for discovering more selective condensate modulators.

More broadly, this study defines a model in which long IDRs encode autoregulatory logic through embedded charge blocks that gate condensation potential. Rather than functioning as passive linkers, long IDRs may act as tunable regulatory modules that encode autoregulatory logic through embedded charge blocks. This mechanism, defined in ASXL1, may extend to other chromatin‐associated proteins where electrostatic heterogeneity is a common feature of long disordered regions. By defining the logic by which charge balance controls condensation, our findings offer a general framework for interpreting how IDR sequence grammar drives nuclear organization and lineage fidelity.

## 4 Experimental Section

### 4.1 Plasmid Construction

#### 4.1.1 Live Imaging Constructs

All full‐length and mutant ASXL1 constructs were C‐terminally tagged with mEGFP via a cleavage‐deficient HA‐TEV‐mutated P2A linker and cloned into the pCS2^+^ vector. Cofactors and ASXL1‐FL/TR constructs were tagged with TagBFP, mCherry or mRuby, each linked via a MYC‐mutated P2A sequence, and cloned into pCS2^+^. To engineer the charge‐mutant ASXL1‐LTR variants (LTR_AtoB, LTR_AtoNon, LTR_BS), we synthesized modified M1M2L2 and M1M2L3 (Sangon Biotech) and inserted each fragment into the pCS2‐N‐M1‐TR1 backbone. TagBFP‐ and mRuby‐containing vectors were provided by Jifeng Fei, and mCherry‐containing vector was provided by Jianchao Li. Constructs were sequence‐validated by Sanger sequencing (Tsingke).

#### 4.1.2 Lentiviral Expression Constructs

The pCDH‐CMV‐MCS‐EF1‐GFP lentiviral expression vector was double‐digested by ClaI and SalI (New England Biolabs). The ASXL1 FL/TR/LTR fragment (CMV‐promoter included), BioID2 sequence, and P2A‐NeoR were then cloned into the digested vector using the Vazyme ClonExpress MultiS One Step Cloning Kit (C113‐02). The BioID2 sequence was amplified from the MCS‐BioID2‐HA vector, which was a gift from Kyle Roux (Addgene plasmid #74224) [40]. pCDH‐puroR vectors were constructed using a similar procedure.

#### 4.1.3 Knock‐in Construct

Guide RNA target sequence was cloned into pSpCas9(BB)‐2A‐GFP (PX458), a gift from Feng Zhang (Addgene plasmid #48138), following the recommended methods [55]. The HaloTag sequence was amplified from pENTR4‐HaloTag (w876‐1), which was a gift from Eric Campeau (Addgene plasmid # 29644). P2A‐NeoR was amplified from pFETCh_Donor (EMM0021), a gift from Eric Mendenhall & Richard M. Myers (Addgene plasmid # 63934) [56]. Homologous arms and inserted fragments were then cloned into pUC19 using the Vazyme ClonExpress MultiS One Step Cloning Kit (C113‐02).

### 4.2 Cell Culture

The 293T (RRID: CVCL_0063), U2OS (RRID: CVCL_0042), HeLa (RRID: CVCL_0030), and K562 (RRID: CVCL_0004) cells were obtained from the Cell Bank/Stem Cell Bank of the Chinese Academy of Sciences, MEG‐01 (RRID: CVCL_0425) from Cellverse (iCell‐h307), and HL‐60 (RRID: CVCL_0002) from Beyotime (C6372). U2OS, 293T, and HeLa cells were maintained in DMEM (Gibco, C12430500BT) supplemented with GlutaMAX (Gibco, 35050061) and 10% FBS (NEWZERUM, FBS‐UE500). K562 and MEG‐01 cells were cultured in RPMI 1640 (Gibco, C11875500BT) with 10% FBS, and HL‐60 cells were grown in IMDM (Gibco, C12440500BT) containing 20% FBS. All cultures were incubated at 37°C with 5% CO_2_ in a humidified incubator. Mycoplasma contamination was tested by PCR using primers Myco‐F (5′‐GAACGGGTGAGTAACACGT‐3′) and Myco‐R (5′‐GGTGTTCTTCCATATCTACGC‐3′), with an annealing temperature of 55 °C for 30 cycles. All cell lines tested negative.

For live‐cell imaging and immunofluorescence, cells were seeded on chambered coverslips (Biosharp, BS‐20‐GJM or Cellvis, D35C4‐20‐1‐N). The following day, cells were transfected using Lipofectamine 3000 (Thermo Scientific, L3000015) according to the manufacturer's instructions.

For drug screening, ASXL1‐TR‐mEGFP U2OS cells were seeded on 96 well glass bottom plates (Cellvis, P96‐1.5P), and treated the following day with compounds from the TargetMol Bioactive Compound Library (Table S5) at a final concentration of 15 µm. After 24 h of treatment, live‐cell imaging was performed using LSM880 confocal microscope equipped with a 40× objective lens.

For neutrophil differentiation, HL‐60 cells were treated with 1 µm Tretinoin for 5 days. For co‐treatment, cells were cultured with 1 µm Tretinoin and 10 µm Tosedostat for 4 days. In both cases, media were refreshed every 2 days.

### 4.3 Stable Line and Knock‐in Cell Line Generation

ASXL1‐EV/FL/TR‐mEGFP‐NeoR, ASXL1‐EV/FL/TR/LTR‐HA‐puroR, and ASXL1‐EV/FL/TR/LTR‐BioID2‐NeoR constructs were cloned into pCDH‐based lentiviral vectors (including the CMV promoter from the pCS2 vector). Lentivirus was produced in 293T cells according to the manufacturer's protocol (Thermo Scientific). Briefly, 293T cells were seeded in lentivirus packaging medium (Opti‐MEM [Gibco, 31985070] containing 5% FBS, 1×GlutaMAX and 1 mm Sodium Pyruvate [Gibco, 11360070]) and incubated overnight. The lentiviral expression vectors and packaging plasmids (pMD2.G and psPAX2) were transfected using Lipofectamine 3000. Six hours post‐transfection, the medium was replaced with fresh lentivirus packaging medium. Virus‐containing supernatants were harvested at 24 and 52 h post‐transfection and passed through a 0.45 µm filter (Biosharp, BS‐PES‐45). Filtered lentivirus was then added to U2OS and HL‐60 cells at a 1:1 ratio of viral supernatant to culture medium, supplemented with 8 µg/mL Polybrene. HL‐60 cells were added to RetroNectin (TaKaRa, #T100A)‐Coated Plates and transduced by spinfection (1800 × rpm, 1.5 h) in the presence of 8 µg/mL polybrene. After a 2‐day transduction, puromycin (Invivogen, ant‐pr‐1; final concentration: 1 µg/mL) or Geneticin (Gibco, 10131035; final concentration: 400 µg/mL for U2OS and 800 µg/mL for HL‐60) was added to select transduced cells. Following selection, clonal cell lines were isolated by serial dilution and expansion, and validated by PCR.

HA/HaloTag‐tagged knock‐in U2OS cell lines were generated via CRISPR/Cas9‐driven homologous recombination as previously described [56]. Guide RNAs (gRNA) were designed around the desired insertion site (100 bp upstream‐insertion site‐100 bp downstream). Three gRNAs were tested for each insertion site, and the one with the highest efficiency was used for knock‐in. Two plasmids were prepared: (1) a CRISPR/Cas9 vector carrying both Cas9 and gRNA. (2) Donor plasmids were constructed by cloning the HA or HaloTag sequence, followed by a NeoR cassette, along with 700–800 bp homology arms, into a pUC19 backbone. These plasmids were co‐transfected into U2OS cells, and the transfected cells were subsequently selected with Geneticin as described above. Knock‐in clones were isolated and validated by genomic PCR and immunofluorescence.

### 4.4 Live‐Cell Imaging

All live‐cell imaging was performed 24–50 h post‐transfections using a Zeiss LSM800 or LSM880 confocal microscope equipped with a 5% CO_2_ incubation chamber and a heat stage set to 37°C (LSM880). Images were captured with a 63× (and 40× for quantitative analysis). Nuclei were labeled by incubating cells with Hoechst 33342 (Thermo Scientific, 33342; 0.2 µg/mL) for approximately 30 min before imaging. Cell membranes were visualized by staining with CellMask (Thermo Scientific, C10045/C10046) for at least 15 min. Phenol red‐free FLUOROBRITE DMEM (Gibco, A1896701) supplemented with 10% FBS was used during confocal imaging.

FRAP was performed on cells expressing mEGFP‐tagged ASXL1‐TR or its mutant variants. Time‐lapse series were acquired over 85 cycles at 0.8 s intervals. Photobleaching was applied using an 80% power 488 nm laser pulse after the fifth frame. For each cell, three regions of interest (ROIs) were defined: the bleached region, a background region, and the total fluorescence area. Fluorescence intensities from 5 individual cells were quantified using ZEN Blue Software and normalized using easyFRAP [57].

For observing fusion events, 293T cells expressing ASXL1‐TR were imaged in time‐lapse mode at 49 s intervals using a Zeiss LSM880 confocal microscope.

### 4.5 Immunofluorescence

Stable U2OS cell lines expressing HA‐tagged ASXL1 were fixed with 4% paraformaldehyde (PFA) in PBS for 10 min at room temperature. After three PBS washes, cells were permeabilized with 0.1% Triton‐X‐100 for 15 min at room temperature and then washed three more times in PBS. Blocking was performed with 5% FBS in PBS for 1 h at room temperature. Next, cells were incubated overnight at 4°C with primary antibodies, Anti‐HA (MBL, M180‐3) or Anti‐ASXL1 (GeneTex, GTX127284), diluted 1:500 in blocking buffer. After four PBS washes, cells were incubated for 2 h at room temperature with Alexa Fluor 594, 488, 647 ‐conjugated donkey anti‐mouse or anti‐rabbit antibodies (1:400 dilution). Samples were washed five times in PBS and mounted with DAPI‐containing mounting medium.

### 4.6 Imaging Analysis

Mean fluorescence intensity per cell was quantified under identical laser settings using ZEN Blue Software. For each cell, we also recorded whether visible condensates were present. To rigorously compare condensate‐forming capacity across groups while controlling for protein expression levels (as indicated by fluorescence intensity), we performed propensity score matching (PSM) with a caliper of 0.2. Cells from both groups were matched by fluorescence intensity using PSM, followed by Fisher's exact test to assess differences in condensate formation.

#### 4.6.1 Line‐Scan Analysis and Scalar Metrics

Line scans were drawn across the nuclear region of interest in ZEN Blue Software along paths intersecting ∼2–3 puncta when present. Traces were lightly smoothed (5‐point running average). Condensation Index (CI) was computed per line as CI = (*I*_peak_—*I*_baseline_)/σ, with *I*_baseline_ the 20th percentile intensity and σ estimated as 1.4826 × MAD. Peak‐to‐Valley contrast (PVR) was PVR = (*I*_peak_‐*I*_valley_)/(*I*_peak_+*I*_valley_).

#### 4.6.2 Co‐Localization Analysis

Pearson's correlation (FIJI/Coloc2) was computed per cell.

We refer to diffraction‐limited spots observed in images as puncta. When these puncta exhibit liquid‐like behavior (e.g., fusion, rapid FRAP recovery) we refer to them as condensates; in figure legends, we keep the morphological term ‘puncta’ and in the main text, we use ‘condensates’ once liquid‐like behavior is established.

### 4.7 Condensed Area/Intensity Fraction Analysis

For each condition, confocal images were acquired under identical laser power, detector gain, pinhole, and exposure settings. Analyses were performed per nucleus on single optical planes to avoid z‐dependent bias. Segmentations and intensities were obtained in FIJI (intensity window 10–50; comparable to ZEISS 27–140, similar to the range used for puncta counting). Within the nuclear ROI, condensed area fraction (Σ segmented foci area / nuclear area) and the condensed intensity fraction (Σ segmented foci integrated fluorescence / total nuclear fluorescence) were quantified.

### 4.8 Acceptor‐Photobleach FRET (pbFRET)

#### 4.8.1 Imaging

U2OS cells expressing mTurquoise2– [M1‐L3/M1‐L2L3]–mNeonGreen cis sensor (or charge‐rewired variants) were fixed by 4% formaldehyde for 10 min and imaged using a Zeiss LSM800 microscope. “Pre‐bleach” donor (mTurquoise2) and acceptor (mNeonGreen) images were acquired, the acceptor was photobleached within a square ROI (488 nm line at high power, 100 iterations), and “post‐bleach” and acceptor images were collected immediately. A same‐sized, unbleached ROI elsewhere in the nucleus served as a donor drift/photobleach control.

#### 4.8.2 ROI Selection and Background

For each cell we defined: (1) Bleach ROI (acceptor‐bleached), (2) Negative ROI (unbleached control), and (3) Background ROI (cell‐free area). Raw mean intensities were measured for donor and acceptor channels pre and post bleach. Background was subtracted separately for each image and channel. *D*_pre_, *D*_post_ = background‐subtracted donor intensities in the bleached ROI before/after bleach; *DN*_pre_, *DN*_post_ = background‐subtracted donor intensities in the unbleached (negative‐control) ROI before/after bleach.

#### 4.8.3 pbFRET Efficiency Calculation

To correct for donor drift or acquisition photobleaching unrelated to FRET, we computed a scalar correction from the unbleached ROI: *b* = *DN*_post_/*DN*_pre_ and corrected the donor intensity in the bleached ROI: *Corr_D*_post_ = *D*_post_/*b*. FRET efficiency for each ROI was computed using the formula: E = 1 – (*D*_pre_/*Corr_D*_post_).

### 4.9 Long Linker IDR Analysis

#### 4.9.1 Long Linker IDR Identification

IDRs in the human proteome were predicted using metapredict [58], a deep‐learning‐based consensus disorder predictor, with the default threshold score set to 0.5. Regions were defined as a long linker IDR if they met all of the following criteria: (1) IDR_START > 0; (2) IDR_END < total protein length; and (3) length > 1000 amino acids (IDR_END—IDR_START + 1). Proteins containing these regions were classified using the PANTHER protein classification system [59]. For proteins listed as “no PANTHER category is assigned”, we assigned functional categories based on either: (1) the classifications of their homologous genes in other species or (2) the classifications of related family members. We refer to the complete “Human IDRome” as all IDRs exceeding 30 residues, as previously described [25], i.e., regions satisfying (IDR_END—IDR_START + 1) > 30. Regions exceeding 1000 residues were designated as long IDRs.

#### 4.9.2 Identification of Pathogenic Variants in llIDR‐Containing Proteins

We obtained variant information for all proteins containing long linker IDRs from Ensembl BioMart [60]. We filtered for pathogenic variants by searching for “pathogenic” in the “Clinical significance” field. Next, we compared the positions of pathogenic variants with the coordinates of each protein's long linker IDR to identify those variants that lie within llIDRs.

#### 4.9.3 IDR Features Analysis

IDR features in ASXL1 and other llIDR proteins were characterized following previously published methods [25]. Specifically, the long linker IDRs, ASXL1‐specific IDR regions, and subdivided llIDRs were analyzed based on 90 sequence features known or hypothesized to be crucial for the function and/or phase separation of disordered regions [24, 61]. Patterning features were analyzed using NARDINI [27], while additional features were assessed using published definitions and scripts [25]. To assess variation in features across different segments of each llIDR, we first split each llIDR into 200‐amino‐acid blocks (if the last segment contained fewer than 30 residues, it was merged into the preceding block). These features were calculated for each segment, standard deviations were computed across segments within each llIDR, and results were visualized as heatmaps. Feature groups shown in Figure 4A: **NARDINI patterning**: NARDINI binary‐patterning z‐scores for residue‐class pairs (pol, hyd, pos, neg, aro, ala, pro, gly). Positive = blocky clustering; negative = well‐mixed; **Amino‐acid fractions**: Per‐residue composition (Frac A … Frac Y). **Grouped composition**: Frac K+R; Frac D+E; Frac Polar; Frac Aliphatic; Frac Aromatic. **Basic/acidic ratios**: log_10_(K/R) and log_10_(E/D) with +1 pseudocounts; higher K/R = lysine‐biased basicity; higher E/D = glutamate‐biased acidity. **Charge & global properties**: Fraction chain‐expanding residues; FCR (fraction charged residues); NCPR (net charge per residue); mean hydrophobicity; disorder‐promoting fraction; isoelectric point (pI); PPII propensity (polyproline II Propensity): Overall polyproline II propensity of IDR. **Residue “block” content**: For each residue (A…Y, excluding W), fraction of the sequence lying within contiguous runs/patches (≥4 occurrences, ≤2 interruptions). **RG‐stretch content**: Fraction within RG‐rich stretches (≥2 “RG” occurrences, ≤2 interruptions), capturing classic Arg‐Gly patches relevant to RNA/chromatin interfaces.

### 4.10 BioID2‐Mass Spectrometry

U2OS cells were stably transduced with lentivirus expressing EV‐BioID2, FL‐BioID2, or TR‐BioID2. Proximity‐dependent labelling (BioID2) was then conducted as previously described [62, 63]. Briefly, U2OS stable cells were cultivated in four 15 cm dishes and treated with 50 µm biotin for 24 h. Cells were washed with cold PBS, resuspended in PBS containing 1 mm EDTA for 10 minutes, and collected by centrifugation. Pelleted cells were then lysed in 1.2 mL of BioID lysis buffer, as previously described [62]. This buffer contained 0.5% Igepal CA‐630, 0.1% SDS, 1.5 mm Na_3_VO_4_, 1 mm PMSF and 1 × protease inhibitor cocktail in HENN buffer (90 mm NaCl, 5 mm EDTA, 50 mm HEPES pH 8.0, 50 mm NaF). Lysates were treated with 1 µL of benzonase (Millipore, E1014) and incubated on ice for 20 min. Then the lysates were sonicated on ice at 60% amplitude for a total of 10 s, delivered in five 2 s bursts with 2 s intervals between bursts. After centrifugation at maximum speed for 10 min at 4°C. Clarified supernatants were incubated overnight at 4°C with NeutrAvidin beads (Thermo Fisher, 29202). The beads were washed three times with wash buffer (0.5% Igepal CA‐630, 1 mm PMSF, 1.5 mm Na_3_VO_4_, 1 mm PMSF, and 1 × protease inhibitor cocktail in HENN buffer) before being washed three more times in ABC buffer (50 mm ammonium bicarbonate, pH 8). On‐bead digestion was performed with trypsin (Promega, V5113) for 18 h, as previously described [63]. The resulting peptides were then dried and submitted for mass spectrometry (MS) analysis. Mass spectrometry data were scored using SAINTexpress (Significance Analysis of INTeractome) software [64], then visualized by ProHits [45].

### 4.11 ChIP‐Seq, CUT&Tag, and ATAC‐Seq Library Preparation

#### 4.11.1 ChIP‐Seq

ChIP‐seq in U2OS BioID stable lines and knock‐in cells was performed as previously described [65]. Briefly, cells were washed with PBS and crosslinked in 1% formaldehyde (Thermo Scientific #28908) for 10 min, then quenched with 125 mm glycine and 0.5% BSA for 5 min. Cells were washed twice with cold 0.5%BSA/PBS and resuspended in Resuspension buffer (9 mm HEPES‐KOH pH7.9, 76.4 mm KCl, 0.9 mm EDTA, 0.5% Igepal CA‐630). The isolated nuclei were pelleted and resuspended in Lysis Buffer (20 mm Tris‐HCl pH 7.5, 150 mm NaCl, 1 mm EDTA, 0.5 mm EGTA, 0.4% Sodium deoxycholate, 0.1% SDS, 1% Igepal CA‐630, 0.5 mm DTT, 1 mm PMSF and 1 × protease inhibitor cocktail), then sonicated for 12 cycles (30 s on/30 s off) using a Bioruptor (Diagenode). The lysates were clarified by centrifugation (20 000 g, 10 min, 4°C), and the supernatants were incubated with the magnetic beads: Pierce Anti‐HA Magnetic Beads (Invitrogen #88836) for ASXL1 or Protein A Mag Sepharose (Cytiva #28951378) with BRD2 antibody (Abcam ab13960). Following a series of washes, chromatin–protein complexes were eluted and reverse‐crosslinked, followed by RNase A and protease K digestion. DNA was purified using AMPure XP (Beckman, A63881), alongside 1% input control. ChIP‐seq libraries were prepared using the NEBNext Ultra II kit (NEB #E7645S) following the manufacturer's instructions.

#### 4.11.2 CUT&Tag

CUT&Tag was performed in ASXL1‐HA U2OS knock‐in cells using the NovoNGS CUT&Tag 4.0 High‐Sensitivity Kit (N259‐YH01) according to the manufacturer's protocol.

#### 4.11.3 ATAC‐Seq

ATAC‐seq in U2OS knock‐in cells was performed using High‐Sensitivity Open Chromatin Profile Kit 2.0 (N248), according to the manufacturer's instructions.

All ChIP‐seq, CUT&Tag, and ATAC‐seq libraries were sequenced on an Illumina NovaSeq platform to generate 2 × 150 bp pair‐end reads.

### 4.12 ChIP‐Seq Data Analysis

Sequencing quality was assessed using FastQC (v0.12.1) [66]. Adapter contamination was trimmed using Trimmomatic (v0.39) [67]. Reads were aligned to the human reference genome (GRCh38) using Bowtie 2 (v2.5.4) [68] under default parameters. Peak calling was performed with MACS2 (v2.2.9.1) [69] using the input control data and default parameters. SAM files were converted to BAM format with Samtools (v1.7) [70]. Differential peaks were identified via the Diffbind Bioconductor package (v3.12.0) [71] with default parameters. BAM files were also converted to BigWig coverage files with deepTools (v3.5.5) [72]. Genomic binding profiles were generated at differential peaks using deepTools ‘computeMatrix’, and visualized with ‘plotHeatmap’.

### 4.13 ATAC‐Seq Data Analysis

Sequencing quality was evaluated using FastQC (v0.12.1) [66]. Adapters were trimmed using Trim_Galore (v0.6.10) [73]. Reads were aligned to GRCh38 with Bowtie 2 (v2.5.4) [68] under default parameters. SAM files were converted to BAM format using Samtools (v1.7) [70], and PCR duplicates were removed with Picard tools. Unmapped, low‐quality, and mitochondrial reads were excluded. Strand‐specific read offsets were applied: +4 bp for forward reads, and −5 bp for reverse reads. BAM files were then converted to BigWig coverage tracks with deepTools (v3.5.5) [72]. Signal profiles at BRD2 differential peaks were computed with deepTools ‘computeMatrix’, and visualized using ‘plotHeatmap’.

#### 4.13.1 Chromatin Accessibility Transition Analysis

Peak calling was performed using MACS2 (v2.2.9.1) with custom parameters: –broad –nomodel –shift ‐37 –extsize 73 –keep‐dup all, using BEDPE‐formatted input derived from paired‐end BAM files [54]. For each cell line (WT, FL, TR, LTR), peaks from two replicates were merged using bedtools ‘merge’ (v2.26.0) [74] with a maximum gap of 100 bp. A unified reference peak set was then generated by merging all condition‐level peak files. For each sample, bedtools ‘intersect’ was used to determine whether each reference peak overlapped a peak in the sample's merged peak file (≥1 bp overlap). Each reference peak was labeled as ‘open’ if an overlap was detected, and ‘closed’ otherwise, yielding a binary accessibility matrix across the four conditions. Each peak was then assigned a four‐character accessibility pattern reflecting its state across WT → FL → TR → LTR (e.g., “CCOC” = closed in WT and FL, open in TR, closed in LTR). The number of peaks corresponding to each unique pattern was quantified. To visualize accessibility transitions, a random subset of 5000 peaks was used to generate an alluvial plot with the ggalluvial R package (0.12.5). Bar plots of the most frequent accessibility transition patterns were created using ggplot2 (3.5.1).

### 4.14 CUT&Tag Data Analysis

Sequencing quality was evaluated using FastQC (v0.12.1) [66]. Adapters were trimmed using Trim_Galore (v0.6.10) [73]. Reads were mapped to GRCh38 with Bowtie 2 (v2.5.4) [68] under default setting. SAM files were converted to BAM format via Samtools (v1.7) [70]. PCR duplicates were removed with Picard tools, and unmapped, low‐quality, or mitochondrial reads were excluded. BAM files were next converted to BigWig coverage files using deepTools (v3.5.5) [72]. Signal profiles at BRD2 differential peaks were calculated with deepTools ‘computeMatrix’, and heatmaps were visualized using ‘plotHeatmap’. Peaks were annotated to the nearest gene using ChIPseeker v1.38.0 [75] (annotatePeak) with promoter/TSS defined as ±1 kb around the TSS and flanking gene information added up to 5 kb. Gene ontology (GO) for biological processes was enriched using the Metascape online tool [76].

### 4.15 Stable Line Identification

#### 4.15.1 PCR

HL‐60 stable cells were collected, and genomic DNA was extracted by incubation in Buffer NP (100 mm Tris‐HCl pH 8.3, 50 mm KCl, 0.3% Tween‐20, 0.3% NP‐40, and 500 µg/mL proteinase K) at 55°C for 5 h, followed by proteinase K inactivation at 95°C for 10 min. The resulting lysates were used directly as templates to detect ASXL1 cDNA and puromycin resistance gene integration by PCR. The following primers were used: ASXL1 genomic DNA: FP: 5’‐ TGAGTTGTACCTTGCTGTCACAGA‐3’; RP: 5’‐ TCCACATCAGCCGTGTCCTC‐3’; ASXL1 cDNA: FP: 5’‐ CTGTGAGTGGTGAAAACGATGTAT‐3’; RP: 5’‐ CTTTTGTTTGTTCGCCTGTGA‐3’; GAPDH genomic DNA: FP: 5’‐ TGCTGCATTCGCCCTCTTAA‐3’; RP: 5’‐ CGCCCAATACGACCAAATCTAA‐3’; puroR: FP: 5’‐ CCGAGTTGAGCGGTTCCC‐3’; RP: 5’‐ AGACCCTTGCCCTGGTGG‐3’.

#### 4.15.2 Western Blot

HL‐60/U2OS stable cells were washed with cold PBS and lysed in 0.5 mL lysis buffer (0.5% Igepal CA‐630, 1 mm PMSF, 1.5 mm Na_3_VO_4_, 1 mm PMSF, and 1 × protease inhibitor cocktail in HENN buffer). Lysates were treated with 1 µL of benzonase (Millipore, E1014) and incubated on ice for 20 min. Sonication was performed on ice at 60% amplitude for 10 s total, delivered in five 2‐second bursts with 2 s intervals. Following centrifugation at maximum speed for 10 min at 4°C, clarified supernatants were incubated overnight at 4°C with Pierce Anti‐HA Magnetic Beads (Invitrogen #88836). Beads were washed three times with lysis buffer (0.5% Igepal CA‐630, 1 mm PMSF, 1.5 mm Na_3_VO_4_, 1 mm PMSF and 1 × protease inhibitor cocktail in HENN buffer). MEG‐01 cells were washed with cold PBS and lysed in RIPA buffer (10 mm Tris‐HCl pH 7.5; 150 mm NaCl; 0.5 mm EDTA; 0.1%SDS; 1% Triton X‐100; 1% Deoxycholate). Lysates were incubated on ice for 30 min. Following centrifugation at maximum speed for 10 min at 4°C. Western blotting was performed according to standard protocols. Primary antibodies: Anti‐HA (MBL, M180‐3), Anti‐ASXL1 (GeneTex, GTX127284), and Anti‐α‐tubulin (NOVUS, NB100‐690). Secondary antibodies: HRP‐conjugated anti‐mouse (Abcam, ab6728). Detection was performed using SuperSignal West Pico PLUS chemiluminescent substrate (Thermo Scientific, 34580).

### 4.16 Drug Screening

ASXL1‐TR‐mEGFP U2OS cells were seeded on 96 well glass bottom plates (Cellvis, P96‐1.5P), and treated the following day with compounds from the TargetMol Bioactive Compound Library (Table S5) at a final concentration of 15 µm. After 24 h of treatment, live‐cell imaging was performed using LSM880 confocal microscope equipped with a 40× objective lens.

### 4.17 Statistical Analysis

All statistical tests were two‐sided with *α* = 0.05. Data are shown as mean ± SD unless stated otherwise. Each “*n*” denotes a single cell (image‐based assays) or a biological replicate as indicated in figure legends. No statistical methods were used to predetermine sample size; all available quality‐controlled cells/replicates were included. Homogeneity of variance (Levene) was assessed to guide test selection. Two groups: two‐tailed Student's *t*‐test (Welch's correction applied automatically if variances were unequal). >2 groups (one factor): one‐way ANOVA. If (Levene *p* ≥ 0.05): Fisher's LSD post‐hoc (variances were equal). If (Levene *p* < 0.05): Dunnett's T3 post‐hoc (variances were unequal). Exact test used, *n*, and *p*‐values (or adjusted *p*‐values for post‐hoc) are reported in each legend. Statistical analyses were performed in SPSS (v25) and R (v4.3.1); ZEISS Blue and FIJI were used for image quantification.

## Author Contributions

X.F. designed the research, performed experiments, analyzed data, generated figures, and wrote the manuscript. W.Z. supervised the study and provided study materials, reagents, instrumentation, and computational resources. Q.L. discussed the results and commented on the manuscript.

## Conflicts of Interest

The authors declare no conflicts of interest.

## Supporting information




**Supporting File 1**: advs73852‐sup‐0001‐SuppMat.docx.

**Supporting File 2**: advs73852‐sup‐0002‐SuppTables.docx.

**Supporting File 3**: advs73852‐sup‐0003‐MovieS1.avi.

**Supporting File 4**: advs73852‐sup‐0004‐MovieS2.avi.

## Data Availability

The data that support the findings of this study are openly available in the Gene Expression Omnibus (GEO) at https://www.ncbi.nlm.nih.gov/geo/query/acc.cgi?acc = GSE297436, reference number 297436.
