# Supplementary material for: The Disordered Region of ASXL1 Acts as an Auto‐Regulator Through Condensation
Source: Adv Sci (Weinh). 2026 Jan 20;13(17):e10999. doi: 10.1002/advs.202510999 (PMC13042807; doi:10.1002/advs.202510999)
Supplement: Supplementary file 1 — Supporting File 1: advs73852‐sup‐0001‐SuppMat.docx. [file ADVS-13-e10999-s004.docx]

Supporting Information

The Disordered Region of ASXL1 Acts as an Auto-regulator through Condensation

Xiao Fang, Qiwei Li, Wenqing Zhang*

X. Fang.

Department of Hematology, Guangzhou First People's Hospital, School of Medicine, South China University of Technology, Guangzhou, China

X. Fang. W. Zhang

Division of Cell, Developmental and Integrative Biology, School of Medicine, South China University of Technology, Guangzhou, China

E-mail: mczhangwq@scut.edu.cn

Q. Li

School of Biology and Biological Engineering, South China University of Technology, Guangzhou, China.

**Supplementary Figure**

**Figure S1. Confirming ASXL1-TR Condensate Formation Across Different Cell Types, Fluorescent Tags, and Tag Orientations.**

(A) Left: Representative confocal images and line-scan quantification of mEGFP-tagged ASXL1-FL and ASXL1-TR in live U2OS, HeLa and K562 cells; Right: Condensation Index (CI) from nuclear line-scans (see Experimental Section) quantifies puncta prominence (U2OS_FL, n = 10; U2OS_TR, n = 10; Hela_FL, n = 7; Hela_TR, n = 7; K562_FL, n = 4; K562_TR, n = 4). Statistics: two-tailed Student’s t-test (*p<0.05, **p<0.01, ***p<0.001; mean ± SD). (B) Left: Representative images and line-scan quantifications of ASXL1-TR fused to mEGFP, mCherry, mRuby, or TagBFP in live 293T cells. Right: CI and peak-to-valley contrast (PVR; see Experimental Section) from nuclear line-scans quantifies puncta prominence (mEGFP, n = 10; mCherry, n = 10; mRuby, n = 10; TagBFP, n = 10). Statistics: one-way ANOVA with post-hoc testing (*p<0.05, **p<0.01, ***p<0.001; mean ± SD). (C) Left: Representative images and line-scan quantification of N-terminally tagged mEGFP-ASXL1-FL or mEGFP-ASXL1-TR in live 293T cells. Right: CI from nuclear line-scans quantifies puncta prominence (mEGFP-FL, n = 6; mEGFP-TR, n = 5; TR-mEGFP, n = 5). Statistics: one-way ANOVA with post-hoc testing (*p<0.05, **p<0.01, ***p<0.001; mean ± SD). (D) Pearson correlation between ASXL1-TR and Hoechst for C-terminally tagged vs N-terminally tagged constructs. Colocalization analyzed by FIJI/Coloc2 (mEGFP-TR, n = 5; TR-mEGFP, n = 9). Statistical analysis: two-tailed Student’s t-test (*p<0.05, **p<0.01, ***p<0.001; error bars, mean ± SD).

**
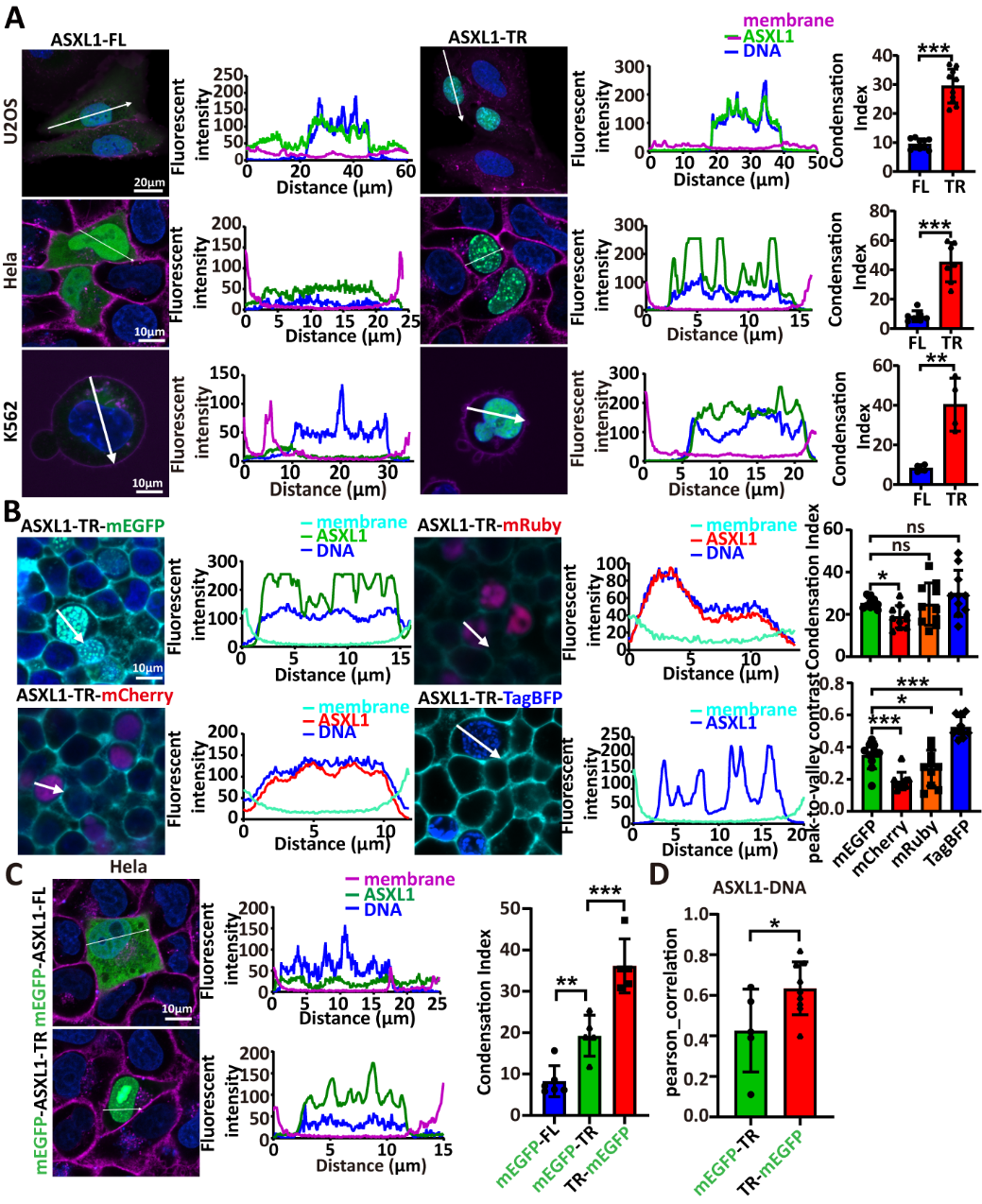
**

**Figure S2. ASXL1-FL retains an intrinsic but normally restrained condensation potential.**


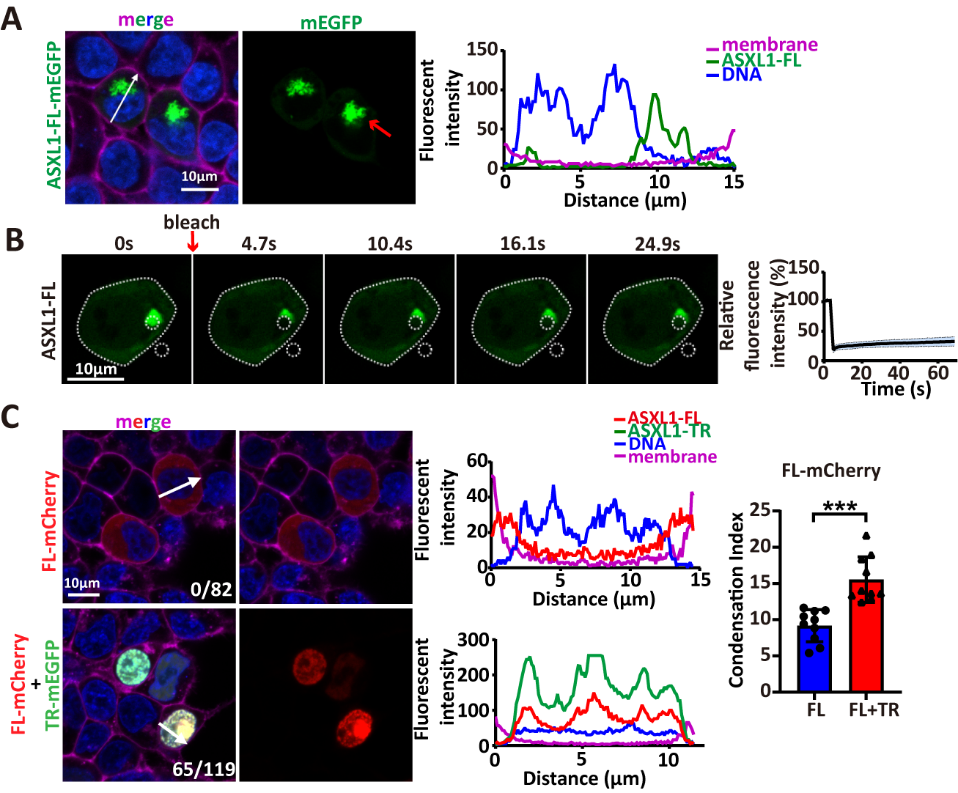
(A) Representative images and line-scan quantification of ASXL1-FL-mEGFP in 293T cells with high expression levels. (B) Representative FRAP images of ASXL1-FL-mEGFP in 293T cells at high expression levels. White dotted circles mark the bleached and background regions; the dashed outline indicates the total-fluorescence ROI. n = 8 biological replicates. (C) Left: Representative images and line-scan quantifications of ASXL1-FL-mCherry expressed without or with ASXL1-TR-mEGFP in live 293T cells. Right: CI from nuclear line-scans (see Experimental Section) quantifies puncta prominence (FL, n = 10; FL + TR, n = 10). Statistics: two-tailed Student’s t-test (*p<0.05, **p<0.01, ***p<0.001; mean ± SD).

**Figure S3. Non-recovering aggregates in C-T7/C-T8 and expression validation of truncated ASXL1.**

(A) Condensate count per cell for TR, N-T3, and N-T5 within an expression-matched bin. Statistics: one-way ANOVA with post-hoc testing (*p<0.05, **p<0.01, ***p<0.001; mean ± SD.) (B) Representative FRAP images for C-T7 and C-T8 (aggregate-like, non-recovering). White dotted circles mark the bleached and background regions; the dashed line outlines the total fluorescence area. n = 5 biological replicates. (C) Condensate count per cell for TR, NL1HM1, and N-M1-TR1 within an expression-matched bin. Statistics as in (A). (D) Representative FRAP images (left) and corresponding fluorescence recovery curves (right) of NL1HM1. White dotted circles mark the bleached and background regions; the dashed line outlines the total fluorescence area. n = 5 biological replicates. (E) Schematic showing endogenous HA knock-in at the ASXL1-FL, TR or LTR loci. (F) Genomic PCR across the insertion site using primers flanking the edit on both sides. (G) Truncated ASXL1 is expressed. Left: Immunoblot of endogenous ASXL1 in MEG-01 (heterozygous G646fsX12) and HA-tagged ASXL1 variants from U2OS knock-in lines after HA immunoprecipitation (IP:HA). Right: Input (pre-IP) lysates from the same U2OS lines. The ASXL1-TR band in U2OS runs slightly above the MEG-01 truncation, consistent with the HA tag and residual P2A linker.


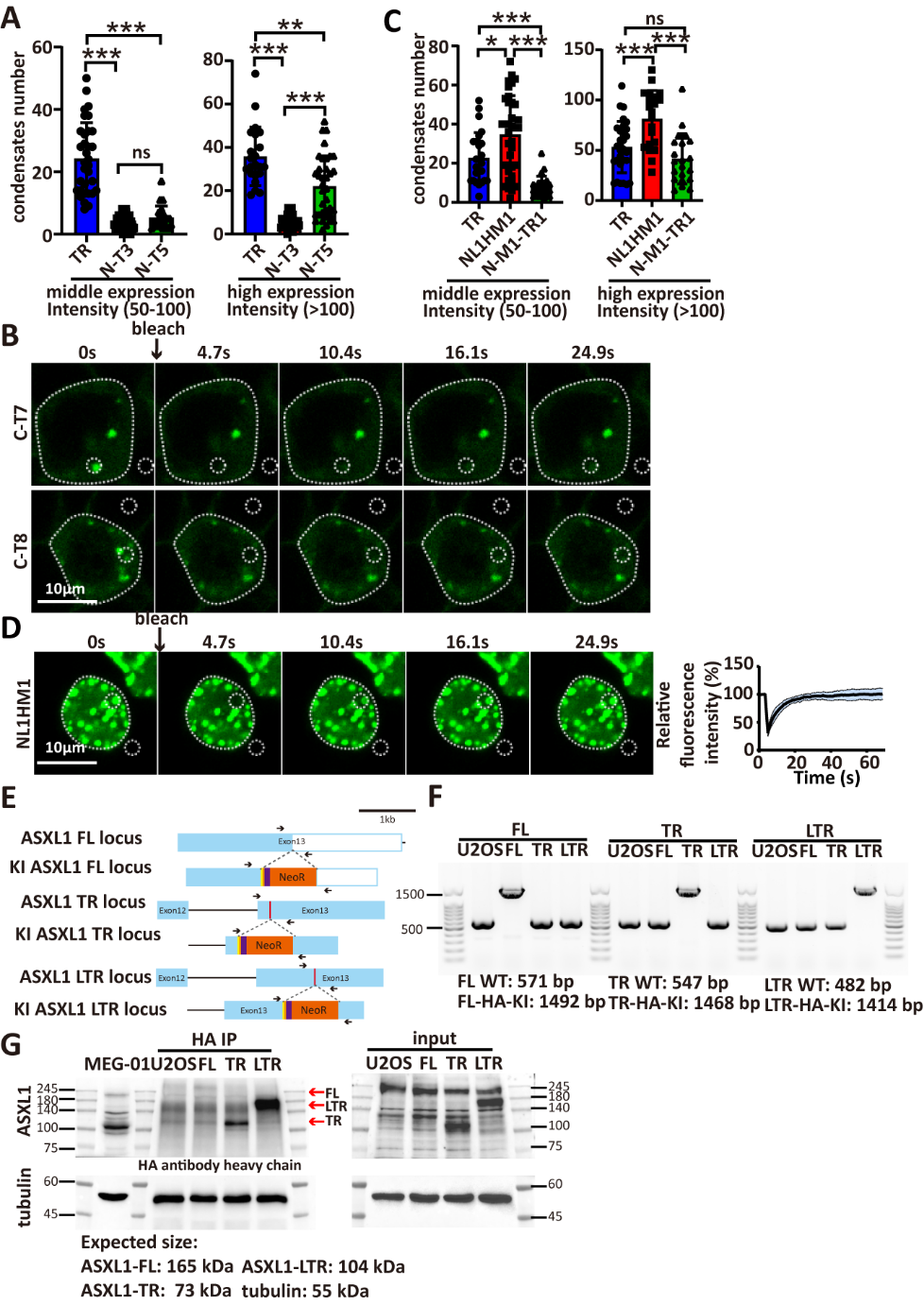


**Figure S4.** **Cis charge pairing within ASXL1’s llIDR provides an autoregulatory brake.**

(A) Condensed area fraction (left) and condensed intensity fraction (right) for LTR mutants (LTR_AtoB, n = 24; LTR_AtoB, n = 15; LTR_BtoNon, n = 13). Statistics: one-way ANOVA with post-hoc testing (*p<0.05, **p<0.01, ***p<0.001; mean ± SD). (B) Representative FRAP images (left) and corresponding fluorescence recovery curves (right) for LTR_AtoB, LTR_AtoNon and LTR_BS. White dotted circles mark the bleached and background regions; the dashed line outlines the total fluorescence area. n = 5 biological replicates. (C) Condensed area fraction (left) and condensed intensity fraction (right) for TR and mutants (TR, n = 32; TR_BtoA, n = 41; TR_BtoNon, n = 42). Statistics: one-way ANOVA with post-hoc testing (*p<0.05, **p<0.01, ***p<0.001; error bars: mean ± SD). (D) Workflow: U2OS cells expressing ASXL1-TR–mEGFP were gently permeabilized (digitonin), then acutely exposed to 300 mM NaCl followed by washout/recovery, and imaged by confocal microscopy. (E) Representative images (left) and line-scan profiles (right) for baseline (−NaCl), +NaCl (300 mM), and recovery after NaCl washout (+NaCl_R). (F) Pearson’s r (ASXL1-TR–mEGFP ↔ Hoechst) for −NaCl (n = 44), +NaCl (n = 46), and +NaCl_R (n = 37). Statistics: one-way ANOVA with post-hoc testing (*p<0.05, **p<0.01, ***p<0.001; mean ± SD). (G) Schematic of mTurquoise2–[M1–L2L3]–mNeonGreen cis FRET sensor and their charge-disrupting variants (AtoB, acidic→basic, AtoNon, acidic→neutral). (H) Representative photobleach FRET images (left) and fluorescent intensity (mTurquoise2 and mNeonGreen) changes between pre-bleach and after-bleach of M1–L2L3, M1–L2L3_ AtoB, and M1–L2L3_ AtoNon. (I) FRET efficiency (see Experimental Section) for [M1–L2L3] sensors (M1-L2L3, n = 10; M1-L2L3_AtoB, n = 10; M1-L2L3_AtoNon, n = 10). Statistics: one-way ANOVA with post-hoc testing (*p<0.05, **p<0.01, ***p<0.001; error bars: mean ± SD).

**
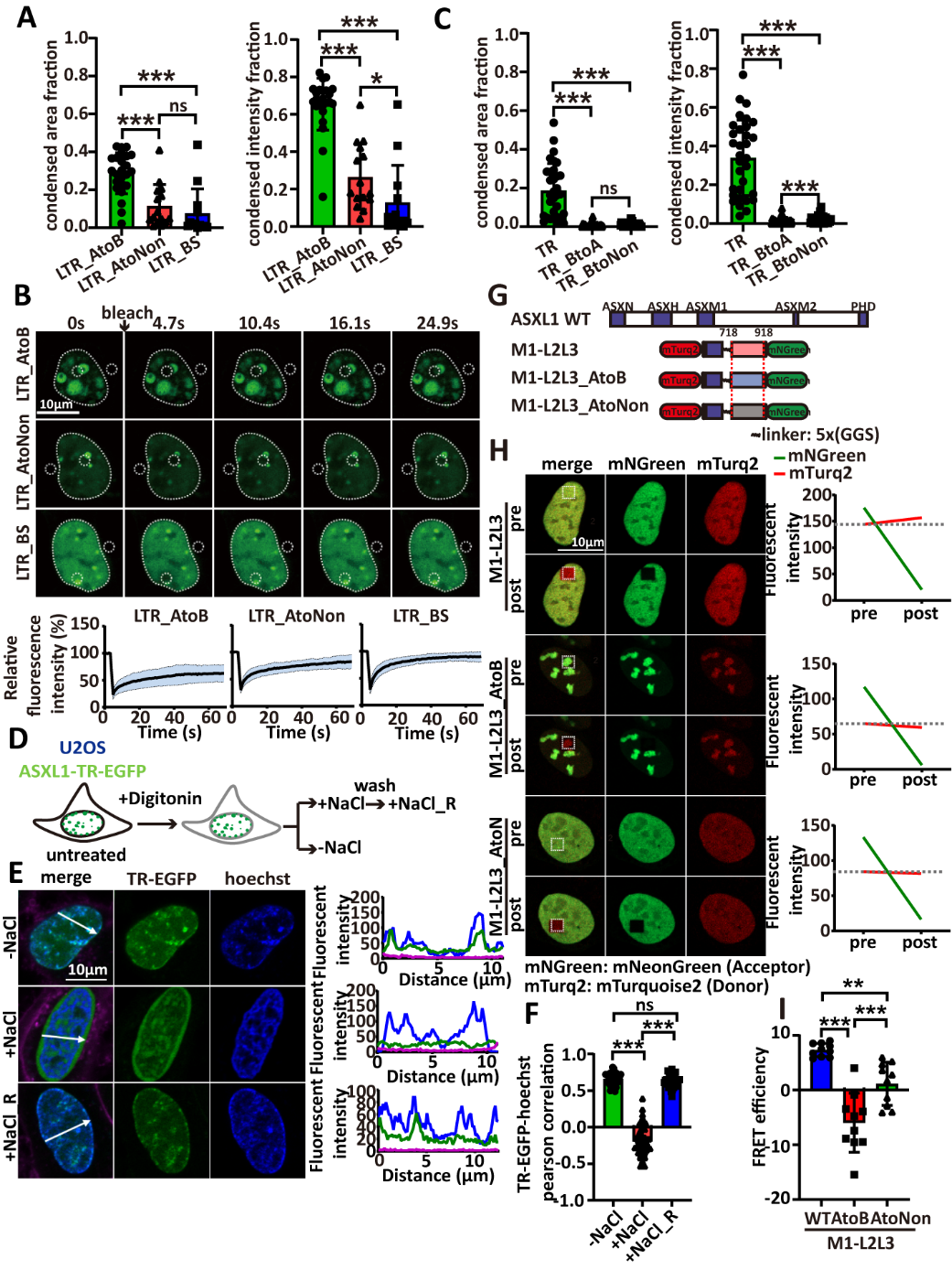
**

**Figure S5. Charge-related features account for the greatest variability.**


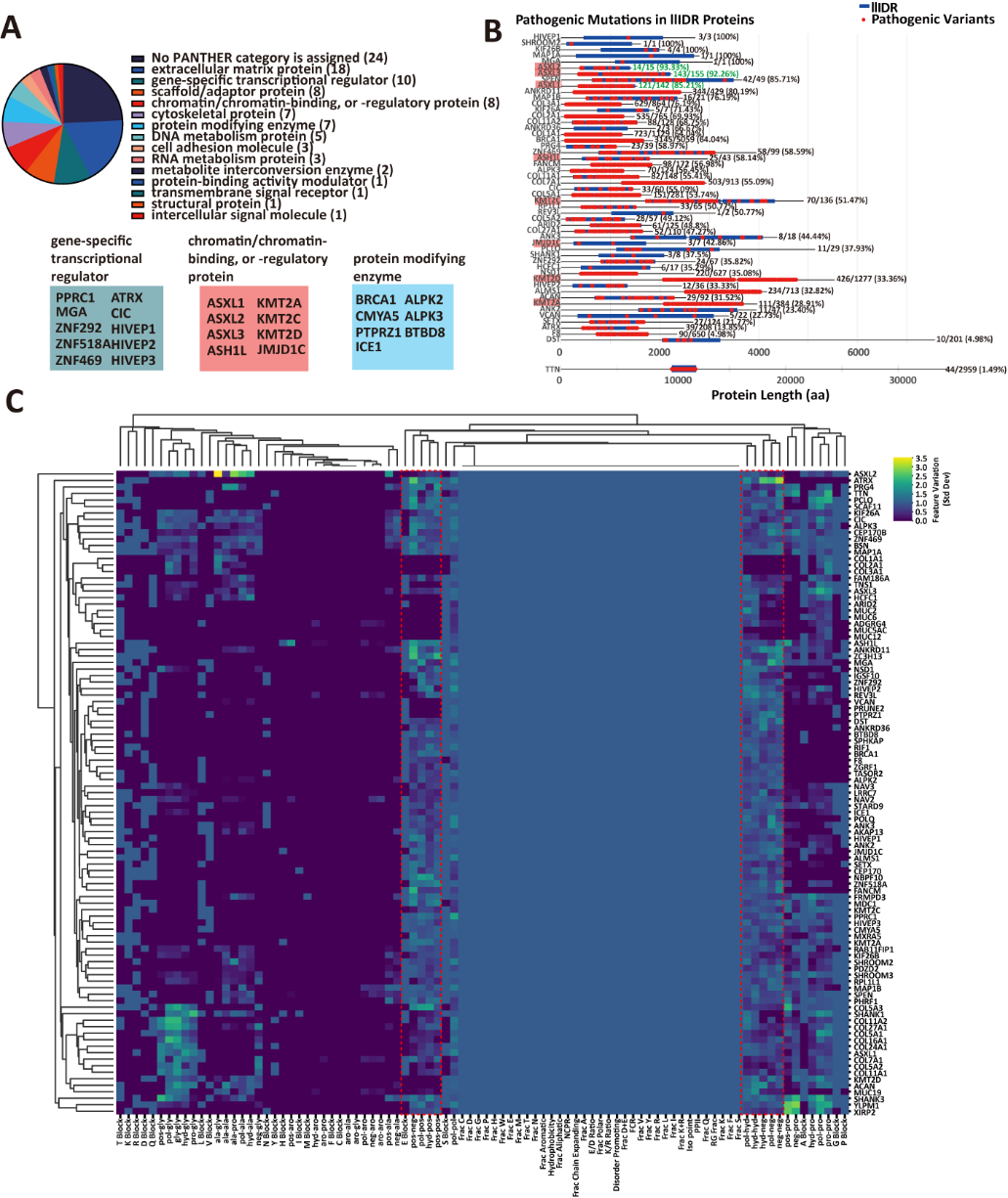
(A) Functional classification of proteins containing long linker IDRs, performed using the PANTHER (Protein Analysis Through Evolutionary Relationship) system. (B) Pathogenic variants mapped within long linker IDRs. Black lines represent total protein length, blue boxes indicate llIDR regions, and red dots mark the positions of pathogenic variants. (C) Each llIDR was divided into 200-amino-acid segments, and sequence features were computed for each segment. The heatmap displays the standard deviation of each feature within individual llIDR.

**Figure S6. The FUS^IDR^ restores LLPS in ASXL1-LTR**


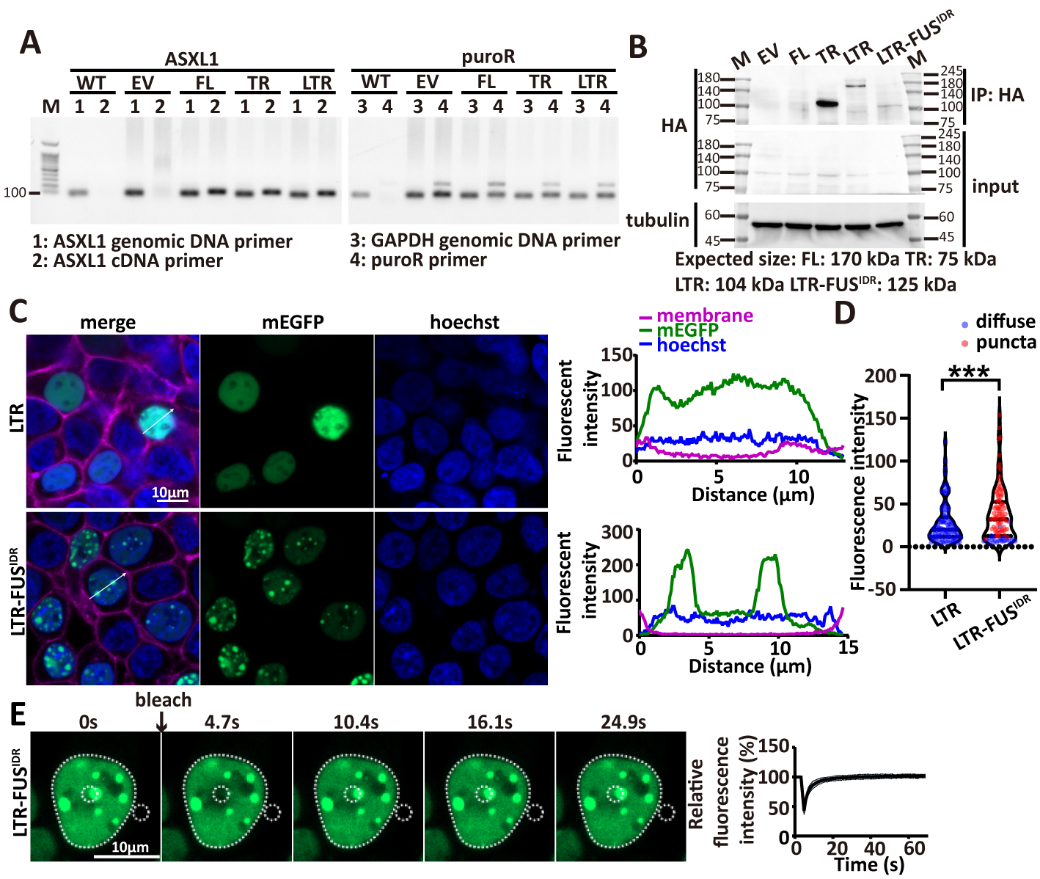
(A) PCR amplification of ASXL1 cDNA and puromycin resistance gene (puroR) from genomic DNA in stable HL-60 lines expressing ASXL1-EV, -FL, -TR, or -LTR constructs. (B) Immunoblot of HA-tagged ASXL1 variants in HL-60 (EV, FL, TR, LTR, LTR-FUS^IDR^); LTR-FUS^IDR^ migrates at the predicted size, whereas TR and LTR run at higher apparent masses. (C) Representative confocal images and line-scan quantifications for ASXL1-LTR-mEGFP and ASXL1-LTR-FUS^IDR^-mEGFP. (D) Violin plot quantifying condensate formation by LTR and LTR-FUS^IDR^ (LTR, n = 114; LTR-FUS^IDR^, n = 111), analyzed using the same method as for the N-T constructs in Figure 2. Statistics: p-value < 2.2 × 10^-16^, *p<0.05, **p<0.01, ***p<0.001; mean ± SD. (D) Representative FRAP images (left) and fluorescence recovery curves (right) for LTR-FUSIDR. White dotted circles denote the bleached and background regions; the dashed line outlines the total fluorescence area. n = 5 biological replicates.

**Figure S7. Generation of BioID2-expressing stable lines.**

(A) Schematic of HA-BioID2-tagged constructs for EV, ASXL1-FL and ASXL1-TR. (B) Western blot analysis of HA-tagged BioID2 fusion proteins in stable lines expressing EV, ASXL1-FL or ASXL1-TR. (C) Representative immunofluorescence images (left) and line-scan quantifications (right) of EV-, ASXL1-FL-, and ASXL1-TR-HA-BioID-expressing U2OS cells. (D) Representative images and line-scan quantifications of ASXL1-TR-mEGFP co-expressed with RRP1-, CT45A6-, ATAD2- and UTP11-TagBFP. (E) Pearson’s r (ASXL1-TR–mEGFP ↔ cofactor-TagBFP) across groups (BRD4, n = 17; BRD2, n = 23; BAP1, n = 22; BRD3, n = 25; RRP1, n = 15; CT45A6, n = 22; ATAD2, n = 22; UTP11, n = 9). Green box showed Pearson’s r (mean) over 0.5. (F) Representative FRAP images (left) and fluorescence recovery curves (right) in cells co-expressing ASXL1-TR with BRD3 or BAP1. White dotted circles mark the bleached and background regions; the dashed line outlines the total fluorescence area. n = 5 biological replicates.


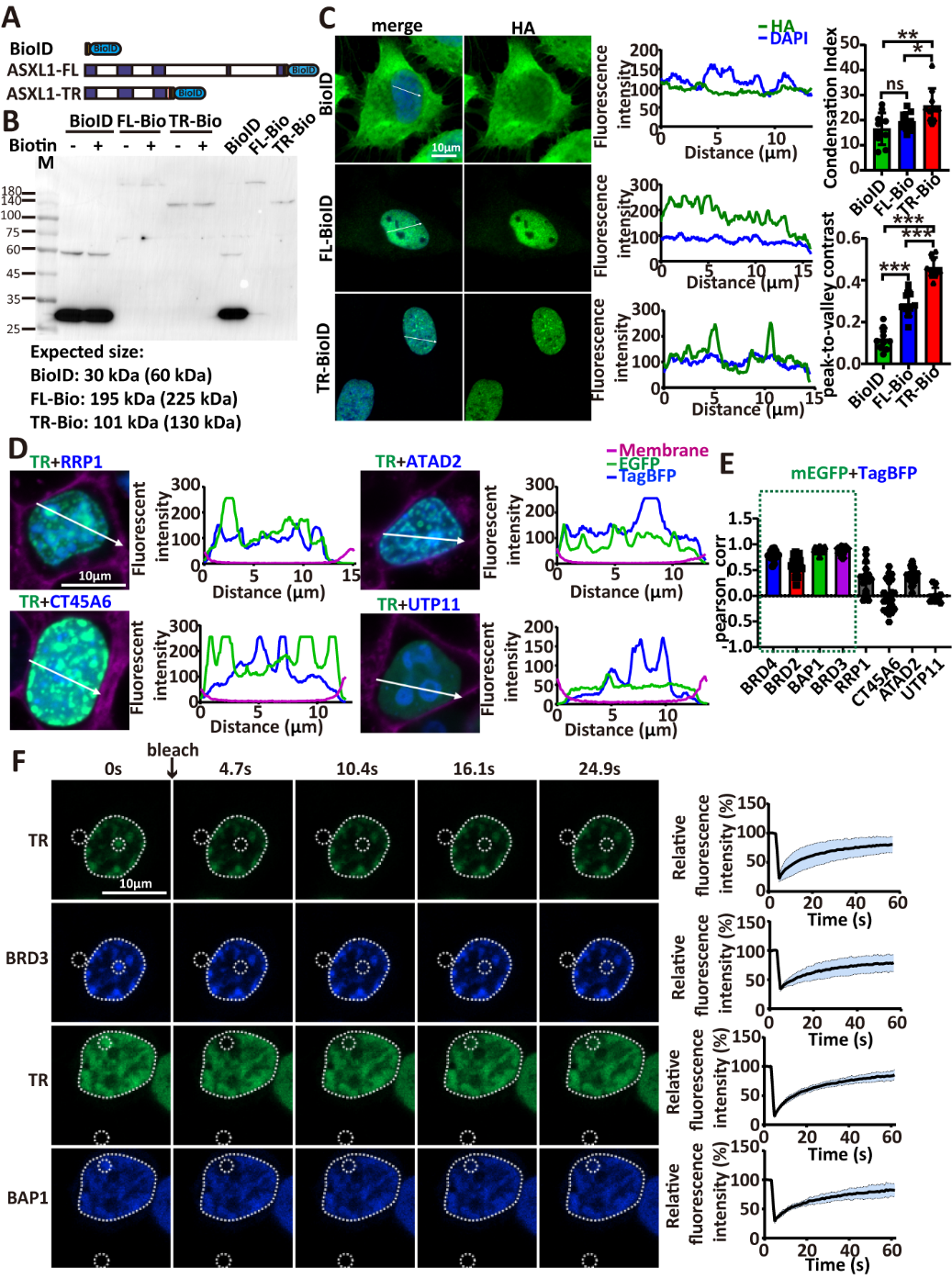


**Figure S8. BRD2 is recruited to ASXL1-TR condensates via its interaction with the ASXM1 domain.**


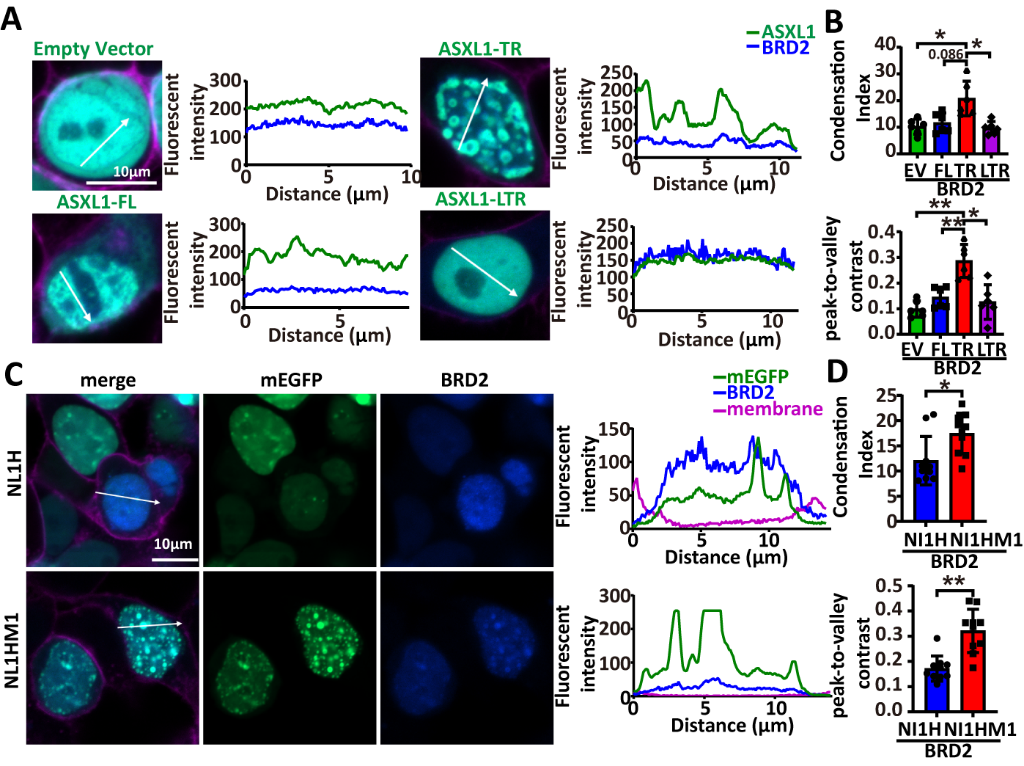
(A) Representative FRAP images (left) and fluorescence recovery curves (right) in cells co-expressing ASXL1-TR with BRD3 or BAP1. White dotted circles mark the bleached and background regions; the dashed line outlines the total fluorescence area. n = 5 biological replicates. (B) Representative images (left) and line-scan quantification (right) of BRD2-TagBFP co-expressed with EV-, ASXL1-FL-, ASXL1-TR-, or ASXL1-LTR-mEGFP. (C) Representative images (left) and line-scan quantifications (right) of BRD2-TagBFP co-expressed with NL1H-mEGFP or NL1HM1-mEGFP.

**Figure S9. ASXL1-TR expression is associated with increased accessibility at a subset of genomic sites.**

(A) IGV view of BRD2 CUT&Tag at the *HOXA7* locus in HL-60 (ASXL1-TR vs ASXL1-LTR). Genome browser snapshots showing BRD2 CUT&Tag signal at the HOXA7 promoter/TSS. Tracks for ASXL1-TR and ASXL1-LTR are displayed on the same y-axis scale (0–10, arbitrary units) with identical track heights (100). (B) BRD2 ChIP-seq profile and ATAC-seq chromatin accessibility at BRD2 differential peaks in ASXL1-TR and ASXL1-LTR knock-in U2OS cells. (C) Alluvial plot showing representative chromatin accessibility transition across U2OS WT, FL, TR, and LTR knock-in cells. A randomly selected subset of 5,000 peaks is shown for clarity. Each flow represents a set of peaks with the same accessibility pattern across the four conditions (Open = red; Closed = blue). (D) Bar plot showing chromatin accessibility transition patterns among peaks that became accessible specifically in ASXL1-TR (i.e., FL → TR, Closed → Open). Patterns are encoded as four-letter codes (C = Closed, O = Open) denoting chromatin states across U2OS WT → FL → TR → LTR order.


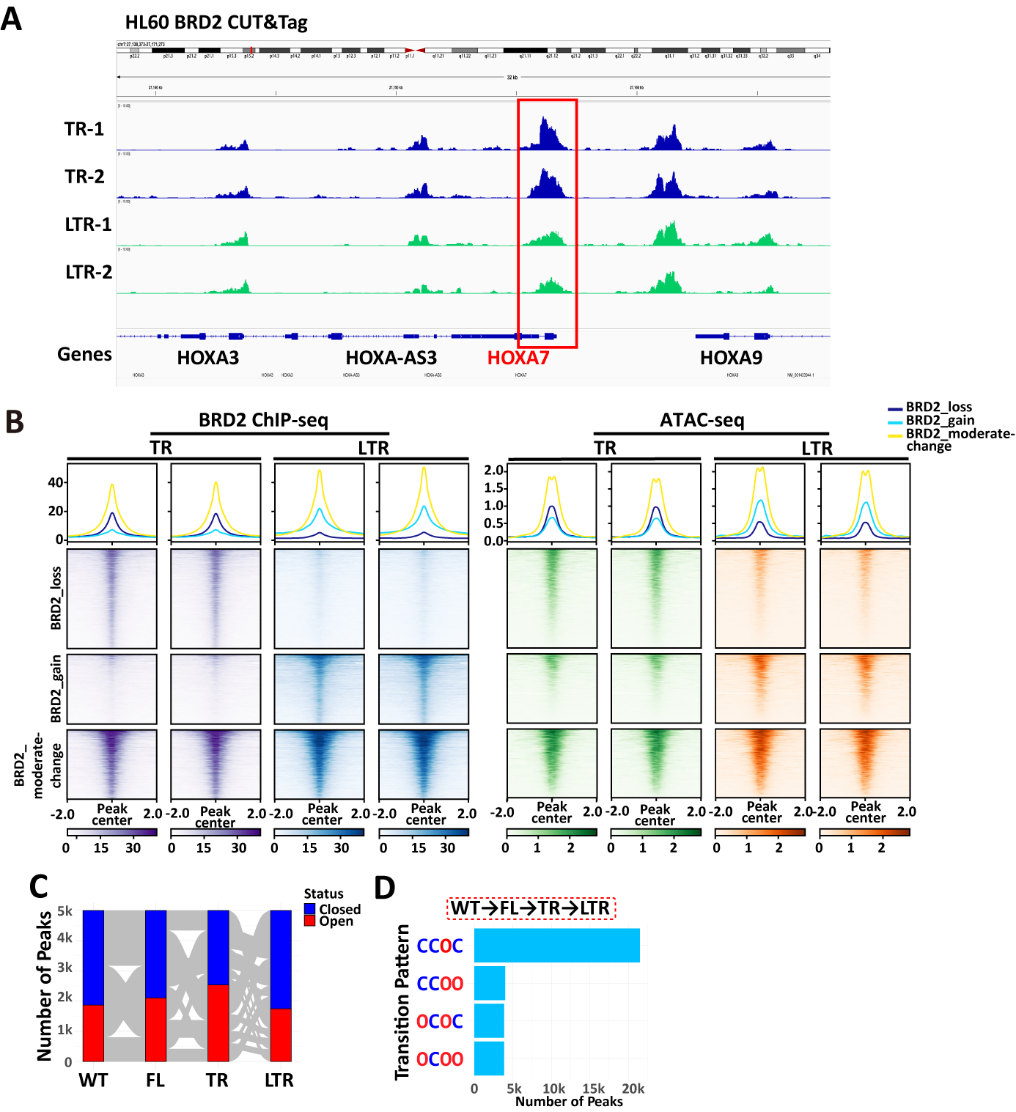


**Figure S10. The aminopeptidase inhibitor Tosedostat reduces ASXL1-TR condensate formation.**


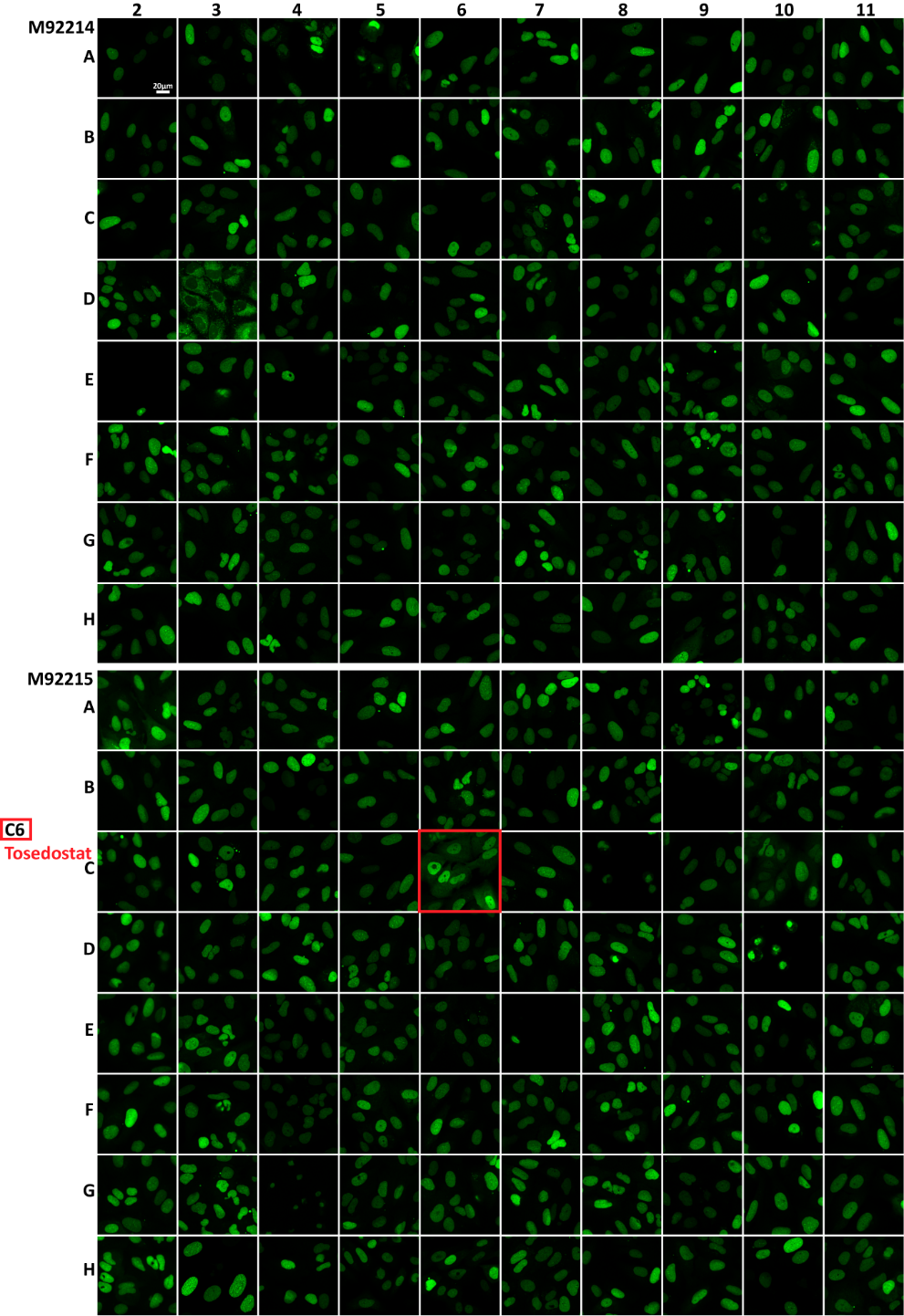
Confocal images of mEGFP-tagged ASXL1-TR in stable U2OS cells following 24-hours treatment with compounds from two screening plates (scale bar: 20 μm; red box: Tosedostat).

**Supplementary Movie Legends**

**Movie S1. Live-cell imaging of dynamic ASXL1-TR condensates.**

Time-lapse confocal imaging of 293T cells expressing ASXL1-TR-mEGFP, showing dynamic condensate behavior, including fusion and fission events. Images were acquired at 49-second intervals using a Zeiss LSM880 confocal microscope under live-cell conditions.

**Movie S2. FRAP analysis of ASXL1-TR condensates.**

Time-lapse series of a FRAP experiment in 293T cells expressing ASXL1-TR-mEGFP. Fluorescence was bleached using an 80% power 488 nm laser pulse after the fifth frame. Recovery was imaged over 85 cycles at 0.8-second intervals using a Zeiss LSM800 confocal microscope.
