# Supplementary material for: The Disordered Region of ASXL1 Acts as an Auto‐Regulator Through Condensation
Source: Adv Sci (Weinh). 2026 Jan 20;13(17):e10999. doi: 10.1002/advs.202510999 (PMC13042807; doi:10.1002/advs.202510999)
Supplement: Supplementary file 2 — Supporting File 2: advs73852‐sup‐0002‐SuppTables.docx. [file ADVS-13-e10999-s001.docx]

**Supplementary Tables**

**Table S1. List of human proteins containing long linker intrinsically disordered regions (llIDRs).**

This table lists all human proteins predicted to contain long linker intrinsically disordered regions (llIDRs). IDRs were identified using metapredict with a disorder threshold of 0.5. Regions were defined as llIDRs if they exceeded 1,000 amino acids in length and were flanked by structured domains (i.e., not N- or C-terminal or full-length IDRs). Each row represents an individual llIDR prediction. Columns include UniProt protein ID, total protein length, gene name, and the start and end coordinates of the predicted llIDR.

| **Protein_ID** | **Gene_Name** | **Protein_Length** | **IDR_START** | **IDR_END** |
| --- | --- | --- | --- | --- |
| A4UGR9 | XIRP2 | 3374 | 1763 | 2767 |
| O14686 | KMT2D | 5537 | 2079 | 3502 |
| O14686 | KMT2D | 5537 | 3598 | 4759 |
| O15018 | PDZD2 | 2839 | 808 | 2571 |
| O60281 | ZNF292 | 2723 | 819 | 1908 |
| O60673 | REV3L | 3130 | 1020 | 2276 |
| O75417 | POLQ | 2590 | 841 | 1888 |
| P00451 | F8 | 2351 | 727 | 1745 |
| P02452 | COL1A1 | 1464 | 94 | 1211 |
| P02458 | COL2A1 | 1487 | 94 | 1249 |
| P02461 | COL3A1 | 1466 | 88 | 1215 |
| P05997 | COL5A2 | 1499 | 97 | 1291 |
| P12107 | COL11A1 | 1806 | 239 | 1574 |
| P13611 | VCAN | 3396 | 335 | 3089 |
| P13942 | COL11A2 | 1736 | 221 | 1542 |
| P15822 | HIVEP1 | 2718 | 455 | 2067 |
| P16112 | ACAN | 2530 | 676 | 2294 |
| P20908 | COL5A1 | 1838 | 244 | 1600 |
| P23471 | PTPRZ1 | 2315 | 409 | 1637 |
| P25940 | COL5A3 | 1745 | 225 | 1499 |
| P31629 | HIVEP2 | 2446 | 232 | 1344 |
| P38398 | BRCA1 | 1863 | 102 | 1647 |
| P46100 | ATRX | 2492 | 426 | 1542 |
| P46821 | MAP1B | 2468 | 515 | 2352 |
| P51610 | HCFC1 | 2035 | 400 | 1808 |
| P78559 | MAP1A | 2803 | 297 | 2689 |
| P98088 | MUC5AC | 5654 | 2216 | 3218 |
| Q01484 | ANK2 | 3957 | 1565 | 3565 |
| Q02388 | COL7A1 | 2944 | 1244 | 2878 |
| Q02817 | MUC2 | 5289 | 1876 | 4434 |
| Q03001 | DST | 7570 | 2042 | 3222 |
| Q03164 | KMT2A | 3969 | 2076 | 3669 |
| Q07092 | COL16A1 | 1604 | 247 | 1432 |
| Q12802 | AKAP13 | 2813 | 255 | 1966 |
| Q12955 | ANK3 | 4377 | 1402 | 2537 |
| Q12955 | ANK3 | 4377 | 2599 | 4088 |
| Q13796 | SHROOM2 | 1616 | 109 | 1433 |
| Q14676 | MDC1 | 2089 | 137 | 1893 |
| Q15652 | JMJD1C | 2540 | 252 | 1721 |
| Q17RW2 | COL24A1 | 1714 | 245 | 1479 |
| Q2M3C7 | SPHKAP | 1700 | 194 | 1609 |
| Q5SW79 | CEP170 | 1584 | 120 | 1284 |
| Q5T1R4 | HIVEP3 | 2406 | 244 | 1601 |
| Q5T200 | ZC3H13 | 1668 | 193 | 1515 |
| Q5UIP0 | RIF1 | 2472 | 970 | 2294 |
| Q5VV67 | PPRC1 | 1664 | 135 | 1526 |
| Q5VWN6 | TASOR2 | 2430 | 805 | 2148 |
| Q5XKL5 | BTBD8 | 1792 | 518 | 1692 |
| Q68CP9 | ARID2 | 1835 | 607 | 1629 |
| Q6UB99 | ANKRD11 | 2663 | 258 | 2399 |
| Q6W4X9 | MUC6 | 2439 | 1163 | 2355 |
| Q6WKZ4 | RAB11FIP1 | 1283 | 129 | 1197 |
| Q6WRI0 | IGSF10 | 2623 | 662 | 1699 |
| Q76L83 | ASXL2 | 1435 | 374 | 1397 |
| Q7Z333 | SETX | 2677 | 597 | 1694 |
| Q7Z5P9 | MUC19 | 8384 | 597 | 8204 |
| Q86TB3 | ALPK2 | 2170 | 99 | 1785 |
| Q86YA3 | ZGRF1 | 2104 | 80 | 1248 |
| Q8IVL0 | NAV3 | 2385 | 203 | 1566 |
| Q8IVL1 | NAV2 | 2488 | 191 | 1688 |
| Q8IWI9 | MGA | 3065 | 1094 | 2407 |
| Q8IWN7 | RP1L1 | 2400 | 412 | 1513 |
| Q8IXJ9 | ASXL1 | 1541 | 361 | 1496 |
| Q8IYD8 | FANCM | 2048 | 624 | 1817 |
| Q8IZC6 | COL27A1 | 1860 | 272 | 1625 |
| Q8IZF6 | ADGRG4 | 3080 | 218 | 2293 |
| Q8N3K9 | CMYA5 | 4069 | 260 | 3513 |
| Q8NEZ4 | KMT2C | 4911 | 1705 | 3232 |
| Q8NEZ4 | KMT2C | 4911 | 3270 | 4325 |
| Q8TCU4 | ALMS1 | 4168 | 2521 | 4032 |
| Q8TF72 | SHROOM3 | 1996 | 109 | 1777 |
| Q8WUY3 | PRUNE2 | 3088 | 356 | 2878 |
| Q8WZ42 | TTN | 34350 | 9859 | 12030 |
| Q92954 | PRG4 | 1404 | 106 | 1110 |
| Q96JG9 | ZNF469 | 3953 | 759 | 3113 |
| Q96L73 | NSD1 | 2696 | 395 | 1543 |
| Q96L96 | ALPK3 | 1705 | 204 | 1269 |
| Q96NW7 | LRRC7 | 1537 | 402 | 1493 |
| Q96RK0 | CIC | 1608 | 271 | 1440 |
| Q96T58 | SPEN | 3664 | 624 | 3493 |
| Q99590 | SCAF11 | 1463 | 267 | 1377 |
| Q9BYB0 | SHANK3 | 1731 | 664 | 1664 |
| Q9C0F0 | ASXL3 | 2248 | 359 | 2210 |
| Q9HBL0 | TNS1 | 1839 | 438 | 1552 |
| Q9NR48 | ASH1L | 2969 | 423 | 1796 |
| Q9NR99 | MXRA5 | 2828 | 665 | 1879 |
| Q9P2P6 | STARD9 | 4700 | 793 | 4335 |
| Q9UKN1 | MUC12 | 5478 | 113 | 5126 |
| Q9ULI4 | KIF26A | 1882 | 729 | 1777 |
| Q9UPA5 | BSN | 3926 | 521 | 2367 |
| Q9Y2F5 | ICE1 | 2266 | 180 | 1910 |
| Q9Y566 | SHANK1 | 2161 | 763 | 2088 |
| Q9Y6V0 | PCLO | 5142 | 1118 | 3210 |
| P49750 | YLPM1 | 2146 | 510 | 1830 |
| Q2KJY2 | KIF26B | 2108 | 802 | 1987 |
| Q9P1Y6 | PHRF1 | 1649 | 234 | 1555 |
| Q9Y4F5 | CEP170B | 1589 | 112 | 1435 |
| A6QL64 | ANKRD36 | 1915 | 257 | 1346 |
| Q5JV73 | FRMPD3 | 1777 | 464 | 1491 |
| Q6AHZ1 | ZNF518A | 1483 | 316 | 1326 |
| A6NE01 | FAM186A | 2351 | 870 | 2100 |
| Q6P3W6 | NBPF10 | 3795 | 400 | 3747 |

**Table S2. Functional classification of llIDR-containing proteins based on PANTHER classification system.**

This table summarizes the functional classification of human proteins containing long linker intrinsically disordered regions (llIDRs). Each protein was annotated using the PANTHER (Protein ANalysis THrough Evolutionary Relationships) classification system, which assigns protein class and subfamily based on sequence and functional similarity. Proteins were grouped into broad functional categories based on their PANTHER protein class annotations. Table columns include UniProt_ID, gene symbol, and protein class.

| **UniProt_ID** | **Gene_Symbol** | **PANTHER_Protein_Class** |
| --- | --- | --- |
| **No PANTHER category is assigned** | | |
| Q8IVL1 | NAV2 | N/A |
| Q92954 | PRG4 | N/A |
| Q2KJY2 | KIF26B | N/A |
| A6QL64 | ANKRD36 | N/A |
| Q5VWN6 | TASOR2 | N/A |
| Q8TCU4 | ALMS1 | N/A |
| Q68CP9 | ARID2 | N/A |
| Q5JV73 | FRMPD3 | N/A |
| Q9ULI4 | KIF26A | N/A |
| A6NE01 | FAM186A | N/A |
| Q5T200 | ZC3H13 | N/A |
| Q96L73 | NSD1 | N/A |
| Q8IVL0 | NAV3 | N/A |
| Q14676 | MDC1 | N/A |
| Q6UB99 | ANKRD11 | N/A |
| Q9P1Y6 | PHRF1 | N/A |
| Q99590 | SCAF11 | N/A |
| Q9Y6V0 | PCLO | N/A |
| P51610 | HCFC1 | N/A |
| Q6P3W6 | NBPF10 | N/A |
| Q9P2P6 | STARD9 | N/A |
| Q12802 | AKAP13 | N/A |
| Q8IWN7 | RP1L1 | N/A |
| Q9UPA5 | BSN | N/A |
| **extracellular matrix protein** | | |
| P20908 | COL5A1 | extracellular matrix structural protein(PC00103) |
| Q02388 | COL7A1 | extracellular matrix structural protein(PC00103) |
| P05997 | COL5A2 | extracellular matrix structural protein(PC00103) |
| Q8IZC6 | COL27A1 | extracellular matrix structural protein(PC00103) |
| P25940 | COL5A3 | extracellular matrix structural protein(PC00103) |
| P16112 | ACAN | extracellular matrix glycoprotein(PC00100) |
| P13611 | VCAN | extracellular matrix glycoprotein(PC00100) |
| P02461 | COL3A1 | extracellular matrix structural protein(PC00103) |
| P13942 | COL11A2 | extracellular matrix structural protein(PC00103) |
| P02458 | COL2A1 | extracellular matrix structural protein(PC00103) |
| Q02817 | MUC2 | extracellular matrix protein(PC00102) |
| P98088 | MUC5AC | extracellular matrix protein(PC00102) |
| Q17RW2 | COL24A1 | extracellular matrix structural protein(PC00103) |
| P12107 | COL11A1 | extracellular matrix structural protein(PC00103) |
| Q07092 | COL16A1 | extracellular matrix structural protein(PC00103) |
| Q6W4X9 | MUC6 | extracellular matrix protein(PC00102) |
| Q7Z5P9 | MUC19 | extracellular matrix protein(PC00102) |
| P02452 | COL1A1 | extracellular matrix structural protein(PC00103) |
| **gene-specific transcriptional regulator** | | |
| Q5VV67 | PPRC1 | transcription cofactor(PC00217) |
| Q8IWI9 | MGA | Rel homology transcription factor(PC00252) |
| O60281 | ZNF292 | C2H2 zinc finger transcription factor(PC00248) |
| Q6AHZ1 | ZNF518A | C2H2 zinc finger transcription factor(PC00248) |
| P46100 | ATRX | DNA-binding transcription factor(PC00218) |
| Q96RK0 | CIC | HMG box transcription factor(PC00024) |
| Q96JG9 | ZNF469 | C2H2 zinc finger transcription factor(PC00248) |
| P15822 | HIVEP1 | C2H2 zinc finger transcription factor(PC00248) |
| Q5T1R4 | HIVEP3 | C2H2 zinc finger transcription factor(PC00248) |
| P31629 | HIVEP2 | C2H2 zinc finger transcription factor(PC00248) |
| **scaffold/adaptor protein** | | |
| Q9Y566 | SHANK1 | scaffold/adaptor protein(PC00226) |
| Q96NW7 | LRRC7 | scaffold/adaptor protein(PC00226) |
| Q5SW79 | CEP170 | scaffold/adaptor protein(PC00226) |
| Q01484 | ANK2 | scaffold/adaptor protein(PC00226) |
| Q2M3C7 | SPHKAP | scaffold/adaptor protein(PC00226) |
| Q9Y4F5 | CEP170B | scaffold/adaptor protein(PC00226) |
| Q9BYB0 | SHANK3 | scaffold/adaptor protein(PC00226) |
| Q12955 | ANK3 | scaffold/adaptor protein(PC00226) |
| **chromatin/chromatin-binding, or -regulatory protein** | | |
| Q8IXJ9 | ASXL1 | chromatin/chromatin-binding, or -regulatory protein(PC00077) |
| Q03164 | KMT2A | histone modifying enzyme(PC00261) |
| Q76L83 | ASXL2 | chromatin/chromatin-binding, or -regulatory protein(PC00077) |
| Q9C0F0 | ASXL3 | chromatin/chromatin-binding, or -regulatory protein(PC00077) |
| Q9NR48 | ASH1L | histone modifying enzyme(PC00261) |
| Q15652 | JMJD1C | histone modifying enzyme(PC00261) |
| O14686 | KMT2D | histone modifying enzyme(PC00261) |
| Q8NEZ4 | KMT2C | histone modifying enzyme(PC00261) |
| **cytoskeletal protein** | | |
| P78559 | MAP1A | non-motor microtubule binding protein(PC00166) |
| Q9HBL0 | TNS1 | non-motor actin binding protein(PC00165) |
| Q13796 | SHROOM2 | actin or actin-binding cytoskeletal protein(PC00041) |
| Q8TF72 | SHROOM3 | actin or actin-binding cytoskeletal protein(PC00041) |
| A4UGR9 | XIRP2 | actin or actin-binding cytoskeletal protein(PC00041) |
| Q03001 | DST | intermediate filament binding protein(PC00130) |
| P46821 | MAP1B | non-motor microtubule binding protein(PC00166) |
| **protein modifying enzyme** | | |
| P38398 | BRCA1 | ubiquitin-protein ligase(PC00234) |
| Q8N3K9 | CMYA5 | ubiquitin-protein ligase(PC00234) |
| P23471 | PTPRZ1 | protein phosphatase(PC00195) |
| Q9Y2F5 | ICE1 | protein modifying enzyme(PC00260) |
| Q96L96 | ALPK3 | non-receptor serine/threonine protein kinase(PC00167) |
| Q86TB3 | ALPK2 | non-receptor serine/threonine protein kinase(PC00167) |
| Q5XKL5 | BTBD8 | ubiquitin-protein ligase(PC00234) |
| **DNA metabolism protein** | | |
| P49750 | YLPM1 | DNA metabolism protein(PC00009) |
| Q5UIP0 | RIF1 | DNA metabolism protein(PC00009) |
| O75417 | POLQ | DNA-directed DNA polymerase(PC00018) |
| Q8IYD8 | FANCM | DNA metabolism protein(PC00009) |
| O60673 | REV3L | DNA-directed DNA polymerase(PC00018) |
| **cell adhesion molecule** | | |
| Q9UKN1 | MUC12 | cell adhesion molecule(PC00069) |
| Q9NR99 | MXRA5 | immunoglobulin superfamily cell adhesion molecule(PC00125) |
| Q6WRI0 | IGSF10 | immunoglobulin superfamily cell adhesion molecule(PC00125) |
| **RNA metabolism protein** | | |
| Q96T58 | SPEN | RNA metabolism protein(PC00031) |
| Q7Z333 | SETX | RNA helicase(PC00032) |
| Q86YA3 | ZGRF1 | RNA helicase(PC00032) |
| **metabolite interconversion enzyme** | | |
| Q8WUY3 | PRUNE2 | phosphodiesterase(PC00185) |
| P00451 | F8 | oxidoreductase(PC00176) |
| **protein-binding activity modulator** | | |
| Q6WKZ4 | RAB11FIP1 | small GTPase(PC00208) |
| **transmembrane signal receptor** | | |
| Q8IZF6 | ADGRG4 | G-protein coupled receptor(PC00021) |
| **structural protein** | | |
| Q8WZ42 | TTN | structural protein(PC00211) |
| **intercellular signal molecule** | | |
| O15018 | PDZD2 | interleukin superfamily(PC00128) |

**Table S3. High-confidence interactors of ASXL1-FL-BioID2 and ASXL1-TR-BioID2.**

This table lists high-confidence proximity interactors of ASXL1-FL and ASXL1-TR identified by BioID2 mass spectrometry in U2OS cells. Proteins were filtered using SAINTexpress based on an average spectral count (AvgSpec ≥ 2) and Bayesian false discovery rate (BFDR ≤ 0.01). Each row represents a prey protein significantly enriched in ASXL1-FL-BioID2 or ASXL1-TR-BioID2 samples relative to EV-BioID2 control. Columns include the bait construct (FL or TR), prey gene symbol, average spectral count across replicates, and BFDR.

| **Bait** | **PreyGene** | **AvgSpec** | **BFDR** |
| --- | --- | --- | --- |
| FL-BioID | MYH10 | 151.5 | 0 |
| FL-BioID | MYH14 | 121 | 0 |
| FL-BioID | ACTN4 | 100.5 | 0 |
| FL-BioID | ACTN1 | 87 | 0 |
| FL-BioID | MY18A | 56 | 0 |
| FL-BioID | MYO1C | 30 | 0.01 |
| FL-BioID | PCCA | 20.5 | 0 |
| FL-BioID | MYO6 | 26 | 0 |
| FL-BioID | ACTB | 236.5 | 0 |
| FL-BioID | DREB | 25 | 0 |
| FL-BioID | MCCB | 17.5 | 0 |
| FL-BioID | FSCN1 | 53 | 0 |
| FL-BioID | TPM1 | 30 | 0 |
| FL-BioID | TPM2 | 20.5 | 0 |
| FL-BioID | MYO1D | 22.5 | 0 |
| FL-BioID | TPM4 | 16.5 | 0 |
| FL-BioID | ACTC | 131 | 0 |
| FL-BioID | LRRF2 | 11.5 | 0 |
| FL-BioID | DDX47 | 10 | 0 |
| FL-BioID | TPM3 | 22.5 | 0 |
| FL-BioID | S2513 | 8.5 | 0 |
| FL-BioID | AFAP1 | 6 | 0 |
| FL-BioID | MYL6B | 12.5 | 0 |
| FL-BioID | GNAI3 | 8 | 0.01 |
| FL-BioID | NEB2 | 12.5 | 0 |
| FL-BioID | ABLM1 | 10 | 0 |
| FL-BioID | ACTBL | 64 | 0 |
| FL-BioID | SEM7A | 7 | 0 |
| FL-BioID | POTEI | 66 | 0 |
| FL-BioID | CHD3 | 7 | 0 |
| FL-BioID | MYO5A | 15 | 0 |
| FL-BioID | RA1L2 | 8.5 | 0 |
| FL-BioID | CA2D1 | 12 | 0 |
| FL-BioID | OBI1 | 6.5 | 0 |
| FL-BioID | ODP2 | 3.5 | 0 |
| FL-BioID | COR2A | 5 | 0 |
| FL-BioID | UHRF1 | 7 | 0 |
| FL-BioID | FLOT2 | 10.5 | 0 |
| FL-BioID | SIPA1 | 6 | 0 |
| FL-BioID | COR2B | 5.5 | 0 |
| FL-BioID | SPTN2 | 6.5 | 0 |
| FL-BioID | LEG8 | 6 | 0 |
| FL-BioID | SMC3 | 5.5 | 0 |
| FL-BioID | AIF1L | 7 | 0 |
| FL-BioID | GNAS1 | 6.5 | 0 |
| FL-BioID | CD109 | 9 | 0 |
| FL-BioID | TMOD1 | 6.5 | 0 |
| FL-BioID | NOP53 | 3.5 | 0.01 |
| FL-BioID | DNJB6 | 3.5 | 0 |
| FL-BioID | TBG1 | 3.5 | 0 |
| FL-BioID | CLPX | 4 | 0 |
| FL-BioID | IMA7 | 3.5 | 0 |
| FL-BioID | XIRP1 | 5.5 | 0 |
| FL-BioID | MAGC1 | 3.5 | 0 |
| FL-BioID | CSRP2 | 2.5 | 0.01 |
| FL-BioID | HYOU1 | 2 | 0.01 |
| FL-BioID | CENPV | 2 | 0.01 |
| FL-BioID | GAPR1 | 3.5 | 0 |
| FL-BioID | DNJB4 | 4 | 0 |
| FL-BioID | SH3L3 | 4 | 0 |
| FL-BioID | NEGR1 | 3 | 0 |
| FL-BioID | SPNXD | 3.5 | 0 |
| FL-BioID | VPP3 | 6 | 0 |
| FL-BioID | C1TM | 3 | 0 |
| FL-BioID | BASI | 3.5 | 0 |
| FL-BioID | THY1 | 3 | 0 |
| FL-BioID | MRT4 | 3.5 | 0 |
| FL-BioID | LRC40 | 4.5 | 0 |
| FL-BioID | NUSAP | 2 | 0.01 |
| FL-BioID | S61A1 | 3 | 0 |
| FL-BioID | UGGG1 | 2 | 0.01 |
| FL-BioID | LG3BP | 3 | 0 |
| FL-BioID | NIPS1 | 2 | 0.01 |
| FL-BioID | NTRI | 3 | 0 |
| FL-BioID | ZP4 | 6 | 0 |
| TR-BioID | ACTB | 246.5 | 0 |
| TR-BioID | MYH14 | 82 | 0 |
| TR-BioID | ACTN1 | 85 | 0 |
| TR-BioID | FSCN1 | 91.5 | 0 |
| TR-BioID | ACTC | 140 | 0 |
| TR-BioID | ACTN4 | 67 | 0 |
| TR-BioID | MY18A | 44 | 0 |
| TR-BioID | ACTBL | 67.5 | 0 |
| TR-BioID | MYO1C | 29 | 0 |
| TR-BioID | TPM1 | 19 | 0 |
| TR-BioID | MCCB | 16.5 | 0 |
| TR-BioID | MYO1D | 25.5 | 0 |
| TR-BioID | MYO6 | 23.5 | 0 |
| TR-BioID | MYO1B | 25.5 | 0 |
| TR-BioID | SYNPO | 13 | 0.01 |
| TR-BioID | DHRS2 | 11 | 0 |
| TR-BioID | BAP1 | 16 | 0.01 |
| TR-BioID | MYO5A | 21.5 | 0 |
| TR-BioID | LRRF2 | 11.5 | 0 |
| TR-BioID | MCM5 | 12 | 0 |
| TR-BioID | TMOD1 | 11.5 | 0 |
| TR-BioID | MYL6B | 12.5 | 0 |
| TR-BioID | BRD4 | 14 | 0 |
| TR-BioID | BRD2 | 10 | 0 |
| TR-BioID | ABLM1 | 9.5 | 0 |
| TR-BioID | BRD3 | 9 | 0 |
| TR-BioID | COR2B | 8 | 0 |
| TR-BioID | MYL1 | 5.5 | 0 |
| TR-BioID | ERF1 | 4.5 | 0.01 |
| TR-BioID | AIF1L | 6 | 0 |
| TR-BioID | SIPA1 | 5.5 | 0 |
| TR-BioID | PP1B | 6 | 0 |
| TR-BioID | COF2 | 4.5 | 0 |
| TR-BioID | ATAD2 | 9.5 | 0 |
| TR-BioID | COR2A | 7 | 0 |
| TR-BioID | GAPR1 | 4 | 0 |
| TR-BioID | XIRP1 | 6 | 0 |
| TR-BioID | CA2D1 | 4 | 0 |
| TR-BioID | LEG8 | 4 | 0 |
| TR-BioID | C1TM | 4 | 0 |
| TR-BioID | HYOU1 | 5.5 | 0 |
| TR-BioID | SH3L2 | 2.5 | 0.01 |
| TR-BioID | NIPS1 | 3 | 0 |
| TR-BioID | MAGC1 | 3 | 0 |
| TR-BioID | ODP2 | 2 | 0.01 |
| TR-BioID | CSRP2 | 3 | 0 |
| TR-BioID | SEM7A | 4.5 | 0 |
| TR-BioID | UHRF1 | 7 | 0 |
| TR-BioID | UGGG1 | 3.5 | 0 |
| TR-BioID | GNAS1 | 4.5 | 0 |
| TR-BioID | SH3L3 | 3 | 0 |
| TR-BioID | CHD7 | 5.5 | 0 |
| TR-BioID | NTRI | 2.5 | 0.01 |
| TR-BioID | RS5 | 3 | 0 |
| TR-BioID | MRT4 | 4 | 0 |
| TR-BioID | ATPK | 2 | 0.01 |
| TR-BioID | RRP1 | 3 | 0 |
| TR-BioID | CNN1 | 2 | 0.01 |
| TR-BioID | SMC3 | 3 | 0 |
| TR-BioID | THY1 | 2.5 | 0.01 |
| TR-BioID | CT456 | 2 | 0.01 |
| TR-BioID | NEGR1 | 2 | 0.01 |
| TR-BioID | CD109 | 4 | 0 |
| TR-BioID | IMA7 | 2.5 | 0.01 |
| TR-BioID | ZP4 | 6.5 | 0 |
| TR-BioID | RT15 | 3.5 | 0.01 |
| TR-BioID | CDK3 | 2.5 | 0.01 |
| TR-BioID | F107B | 2 | 0.01 |
| TR-BioID | UTP11 | 2 | 0.01 |

**Table S4. Relative spectral counts comparing ASXL1-FL-BioID2 and ASXL1-TR-BioID2.**

This table compares the relative enrichment of selected shared proximity interactors between ASXL1-FL-BioID2 and ASXL1-TR-BioID2 in U2OS cells. The proteins included here were filtered from the high-confidence interactors identified in Supplementary Table 3. Values represent normalized spectral counts (relative enrichment scores) for each prey protein, scaled from 0 to 1 within each bait condition. A value of 1 corresponds to the highest observed enrichment in that dataset. This comparison highlights proteins preferentially enriched in either ASXL1-FL or ASXL1-TR condensates.

|  | **FL-BioID** | **TR-BioID** |
| --- | --- | --- |
| ACTB | 0.96 | 1 |
| MYH10 | 1 | 0.8 |
| ACTC | 0.94 | 1 |
| MYH14 | 1 | 0.68 |
| ACTN1 | 1 | 0.98 |
| ACTN4 | 1 | 0.67 |
| FSCN1 | 0.58 | 1 |
| ACTBL | 0.95 | 1 |
| MY18A | 1 | 0.79 |
| POTEI | 1 | 0.61 |
| SPNXD | 1 | 0 |
| DNJB4 | 1 | 0 |
| AFAP1 | 1 | 0 |
| OBI1 | 1 | 0 |
| RA1L2 | 1 | 0 |
| CHD3 | 1 | 0.36 |
| SMC3 | 1 | 0.55 |
| LEG8 | 1 | 0.67 |
| GNAS1 | 1 | 0.69 |
| SEM7A | 1 | 0.64 |
| GNAI3 | 1 | 0.75 |
| AIF1L | 1 | 0.86 |
| UHRF1 | 1 | 1 |
| SIPA1 | 1 | 0.92 |
| ZP4 | 0.92 | 1 |
| XIRP1 | 0.92 | 1 |
| COR2A | 0.71 | 1 |
| COR2B | 0.69 | 1 |
| DDX47 | 1 | 0.55 |
| NEB2 | 1 | 0.44 |
| CA2D1 | 1 | 0.33 |
| CD109 | 1 | 0.44 |
| ABLM1 | 1 | 0.95 |
| LRRF2 | 1 | 1 |
| MYL6B | 1 | 1 |
| SYNPO | 0.58 | 1 |
| MCM5 | 0.54 | 1 |
| TMOD1 | 0.57 | 1 |
| DHRS2 | 0.5 | 1 |
| BAP1 | 0.16 | 1 |
| ATAD2 | 0.11 | 1 |
| BRD3 | 0.06 | 1 |
| TPM4 | 1 | 0.64 |
| PCCA | 1 | 0.54 |
| TPM3 | 1 | 0.62 |
| TPM2 | 1 | 0.71 |
| MCCB | 1 | 0.94 |
| MYO5A | 0.7 | 1 |
| MYO1B | 0.65 | 1 |
| MYO1C | 1 | 0.97 |
| MYO6 | 1 | 0.9 |
| MYO1D | 0.88 | 1 |
| TPM1 | 1 | 0.63 |
| DREB | 1 | 0.72 |
| RS5 | 0.33 | 1 |
| UTP11 | 0.5 | 1 |
| CNN1 | 0.5 | 1 |
| ATPK | 0.75 | 1 |
| NUSAP | 1 | 0.75 |
| SH3L2 | 0.8 | 1 |
| NIPS1 | 0.67 | 1 |
| UGGG1 | 0.57 | 1 |
| CSRP2 | 0.83 | 1 |
| HYOU1 | 0.36 | 1 |
| COF2 | 0.33 | 1 |
| FLOT2 | 1 | 0.19 |
| S2513 | 1 | 0.18 |
| SPTN2 | 1 | 0.23 |
| VPP3 | 1 | 0.25 |
| TBG1 | 1 | 0.43 |
| BASI | 1 | 0.43 |
| CLPX | 1 | 0.38 |
| LRC40 | 1 | 0.44 |
| ODP2 | 1 | 0.57 |
| NEGR1 | 1 | 0.67 |
| NTRI | 1 | 0.83 |
| THY1 | 1 | 0.83 |
| IMA7 | 1 | 0.71 |
| MAGC1 | 1 | 0.86 |
| SH3L3 | 1 | 0.75 |
| RT15 | 0.86 | 1 |
| C1TM | 0.75 | 1 |
| MRT4 | 0.88 | 1 |
| GAPR1 | 0.88 | 1 |
| ERF1 | 0.89 | 1 |
| PP1B | 0.67 | 1 |
| DNJB6 | 1 | 0.29 |
| NOP53 | 1 | 0.29 |
| S61A1 | 1 | 0.33 |
| LG3BP | 1 | 0.17 |
| CENPV | 1 | 0.25 |
| CT456 | 0 | 1 |
| F107B | 0 | 1 |
| CDK3 | 0 | 1 |
| RRP1 | 0 | 1 |
| MYL1 | 0 | 1 |
| CHD7 | 0 | 1 |
| BRD2 | 0 | 1 |
| BRD4 | 0 | 1 |

**Table S5. Compounds from the TargetMol Bioactive Compound Library used in ASXL1-TR condensate screening.**

This table lists the compounds screened in the primary chemical screen for ASXL1-TR condensate disruption. Compounds were sourced from the TargetMol Bioactive Compound Library and dispensed in 96-well plate format (plates M92214 and M92215). Each entry includes the compound name and CAS number, with empty wells indicated where applicable. Screening was performed in a U2OS cell line stably expressing ASXL1-TR-mEGFP, and condensate formation was assessed by live-cell imaging after 24-hour treatment.

**Table S6. Key resources used in this study.**

This table provides a comprehensive list of key reagents and resources used throughout the study, including antibodies, chemicals, peptides, recombinant proteins, cell lines, constructs, media, and commercial assay kits.

| **Reagent or Resource** | **Source** | **Identifier** |
| --- | --- | --- |
| **Antibodies** |  |  |
| HA (Mouse) | MBL | M180-3 |
| H3K9me3 (Rabbit) | Abcam | ab8898 |
| BRD2 (Rabbit) (for Immunofluorescence) | proteintech | 22236-1-AP |
| BRD2 (Rabbit) (for ChIP-seq) | Abcam | ab13960 |
| a-tubulin (Mouse) | NOVUS | NB100-690 |
| FITC-conjugated CD11b | Elabscience | E-AB-F1081C-50T |
| APC-conjugated CD14 | Elabscience | E-AB-F1209E-20T |
|  |  |  |
| **Chamicals, peptides, and recombinant proteins** |  |  |
| Tretinoin | Selleck | S1653 |
| Tosedostat | TargetMol | T6301 |
| Puromycin | Invivogen | ant-pr-1 |
| Geneticin | Gibco | 10131035 |
| RetroNectin | TaKaRa | #T100A |
| formaldehyde | Thermo Scientific | #28908 |
| CellMask™ Plasma Membrane Stains | Thermo Scientific | C10045/C10046 |
| Hoechst 33342 | Thermo Scientific | 33342 |
| benzonase | Millipore | E1014 |
| Biotin | Sigma | B4639-100MG |
| NeutrAvidin beads | Thermo Fisher | 29202 |
| Pierce™ Anti-HA Magnetic Beads | Invitrogen | #88836 |
| Protein A Mag Sepharose | Cytiva | #28951378 |
|  |  |  |
| **Cell lines** |  |  |
| 293T | Cell Bank/Stem Cell Bank of the Chinese Academy of Sciences | SCSP-502 |
| U2OS | Cell Bank/Stem Cell Bank of the Chinese Academy of Sciences | SCSP-5030 |
| K562 | Cell Bank/Stem Cell Bank of the Chinese Academy of Sciences | SCSP-5054 |
| HL-60 | Beyotime | C6372 |
|  |  |  |
| **Cell Culture Media** |  |  |
| DMEM | Gibco | C12430500BT |
| RPMI 1640 | Gibco | C11875500BT |
| IMDM | Gibco | C12440500BT |
| GlutaMAX | Gibco | 35050061 |
|  |  |  |
| **Critical Commercial Assays** |  |  |
| ClonExpress MultiS One Step Cloning Kit | Vazyme | C113-02 |
| NEBNext Ultra II kit | New England Biolabs | #E7645S |
| NovoNGS® CUT&Tag 4.0 High-Sensitivity Kit | Novoprotein | N259-YH01 |
| High-Sensitivity Open Chromatin Profile Kit 2.0 | Novoprotein | N248 |
| SuperSignal™ West Pico PLUS chemiluminescent substrate | Thermo Scientific | 34580 |
|  |  |  |
| **Constructs** |  |  |
| pSpCas9(BB)-2A-GFP (PX458) | Addgene | #48138 |
| pMD2.G | Addgene | #12259 |
| psPAX2 | Addgene | #12260 |
| pCS2-ASXL1-FL-HA-TEV-mP2A-mEGFP | This study | N/A |
| pCS2-ASXL1-TR-HA-TEV-mP2A-mEGFP | This study | N/A |
| pCS2-ASXL1-TR-MYC-mP2A-mCherry | This study | N/A |
| pCS2-ASXL1-TR-MYC-mP2A-TagBFP | This study | N/A |
| pCS2-ASXL1-TR-MYC-mP2A-mRuby | This study | N/A |
| pCS2-ASXL1-FL-MYC-mP2A-mCherry | This study | N/A |
| pCS2-mEGFP-mP2A-MYC-ASXL1-FL | This study | N/A |
| pCS2-mEGFP-mP2A-MYC-ASXL1-TR | This study | N/A |
| ASXL1-FL-HA-P2A-NeoR-Donor | This study | N/A |
| ASXL1-TR-HA-P2A-NeoR-Donor | This study | N/A |
| ASXL1-LTR-HA-P2A-NeoR-Donor | This study | N/A |
| ASXL1-FL-HA-P2A-NeoR-Donor | This study | N/A |
| ASXL1-TR-HaloTag-P2A-NeoR-Donor | This study | N/A |
| ASXL1-LTR-HaloTag-P2A-NeoR-Donor | This study | N/A |
| pCS2-N-T1-HA-TEV-mP2A-mEGFP | This study | N/A |
| pCS2-N-T2-HA-TEV-mP2A-mEGFP | This study | N/A |
| pCS2-N-T3-HA-TEV-mP2A-mEGFP | This study | N/A |
| pCS2-N-T4-HA-TEV-mP2A-mEGFP | This study | N/A |
| pCS2-N-T5-HA-TEV-mP2A-mEGFP | This study | N/A |
| pCS2-N-T6-HA-TEV-mP2A-mEGFP | This study | N/A |
| pCS2-N-T7-HA-TEV-mP2A-mEGFP | This study | N/A |
| pCS2-N-T8-HA-TEV-mP2A-mEGFP | This study | N/A |
| pCS2-C-T1-HA-TEV-mP2A-mEGFP | This study | N/A |
| pCS2-C-T2-HA-TEV-mP2A-mEGFP | This study | N/A |
| pCS2-C-T3-HA-TEV-mP2A-mEGFP | This study | N/A |
| pCS2-C-T4-HA-TEV-mP2A-mEGFP | This study | N/A |
| pCS2-C-T5-HA-TEV-mP2A-mEGFP | This study | N/A |
| pCS2-C-T6-HA-TEV-mP2A-mEGFP | This study | N/A |
| pCS2-C-T7-HA-TEV-mP2A-mEGFP | This study | N/A |
| pCS2-C-T8-HA-TEV-mP2A-mEGFP | This study | N/A |
| pCS2-L1HL2M1-HA-TEV-mP2A-mEGFP | This study | N/A |
| pCS2-NHL2M1-HA-TEV-mP2A-mEGFP | This study | N/A |
| pCS2-NL1L2M1-HA-TEV-mP2A-mEGFP | This study | N/A |
| pCS2-NL1HM1-HA-TEV-mP2A-mEGFP | This study | N/A |
| pCS2-N-M1-TR1-HA-TEV-mP2A-mEGFP | This study | N/A |
| pCS2-N-M1-TR2-HA-TEV-mP2A-mEGFP | This study | N/A |
| pCS2-N-M1-TR3-HA-TEV-mP2A-mEGFP | This study | N/A |
| pCS2-N-M1-TR4-HA-TEV-mP2A-mEGFP | This study | N/A |
| pCS2-LTR_AtoB-HA-TEV-mP2A-mEGFP | This study | N/A |
| pCS2-LTR_AtoNon-HA-TEV-mP2A-mEGFP | This study | N/A |
| pCS2-LTR_BS-HA-TEV-mP2A-mEGFP | This study | N/A |
| pCS2-LTR_FUS^IDR^-HA-TEV-mP2A-mEGFP | This study | N/A |
| pCDH-ASXL1-FL-HA-TEV-P2A-mEGFP | This study | N/A |
| pCDH-ASXL1-TR-HA-TEV-P2A-mEGFP | This study | N/A |
| pCDH-ASXL1-FL-HA-TEV-mP2A-mEGFP | This study | N/A |
| pCDH-ASXL1-TR-HA-TEV-mP2A-mEGFP | This study | N/A |
| pCDH-ASXL1-FL-HA-TEV-P2A-puroR | This study | N/A |
| pCDH-ASXL1-TR-HA-TEV-P2A-puroR | This study | N/A |
| pCDH-ASXL1-LTR-HA-TEV-P2A-PuroR | This study | N/A |
| pCDH-ASXL1-FL-BioID2-HA-P2A-NeoR | This study | N/A |
| pCDH-ASXL1-TR-BioID2-HA-P2A-NeoR | This study | N/A |
| pCDH-ASXL1-EV-HA-TEV-P2A-puroR | This study | N/A |
| pCDH-ASXL1-EV-BioID2-HA-P2A-NeoR | This study | N/A |
|  |  |  |
| **Others** |  |  |
| Lipofectamine 3000 | ThermoFisher | L3000015 |
| AMPure XP | Beckman Coulter | A63881 |
| DAPI-containing mounting medium | Biosharp | BL701A |
